# Medicinal Plants and Their Bioactive Phytochemicals as Emerging Therapeutic Strategies for Alzheimer’s Disease: An Integrative Review of Preclinical and Clinical Evidence

**DOI:** 10.1155/sci5/6124916

**Published:** 2026-06-30

**Authors:** Nawfal Hasan Siam, Najibah Nasrin, Sharmily Saiyara, Hridoy Saha, Debashis Paul Deb, Johirul Islam

**Affiliations:** ^1^ Department of Pharmacy, School of Pharmacy and Public Health, Independent University, Bangladesh (IUB), Bashundhara, Dhaka, 1229, Bangladesh; ^2^ Department of Pharmacy, Faculty of Health and Life Sciences, Daffodil International University (DIU), Birulia, Dhaka, 1216, Bangladesh, daffodilvarsity.edu.bd; ^3^ Department of Pharmacy, School of Pharmacy, University of Asia Pacific (UAP), 74/A, Green Road, Dhaka, 1205, Bangladesh; ^4^ Department of Pharmacy, Faculty of Life and Earth Sciences, Jagannath University, 9-10, Chittaranjan Avenue, Dhaka, 1100, Bangladesh, jnu.ac.bd

**Keywords:** Alzheimer’s disease, amyloid and tau pathology, bioactive phytochemicals, cognitive impairment, medicinal plants, neuroprotection

## Abstract

Alzheimer’s disease (AD) is a progressive neurodegenerative disorder characterized by *β-amyloid* deposition, tau hyperphosphorylation, mitochondrial dysfunction, oxidative stress, neuroinflammation, and blood–brain barrier disruption, collectively leading to widespread cortical and subcortical atrophy. Current FDA‐approved pharmacotherapies, including acetylcholinesterase inhibitors and memantine, provide only modest symptomatic relief and fail to halt disease progression, underscoring the urgent need for alternative therapeutic approaches. Growing evidence highlights medicinal plants and their bioactive phytoconstituents as promising candidates for AD prevention and treatment because of their multitarget mechanisms, favorable safety profiles, and long history of traditional use. This review synthesizes extensive in vitro, in vivo, and clinical studies demonstrating the neuroprotective potential of plant extracts and isolated compounds that exert antioxidant, anti‐inflammatory, antiamyloidogenic, anti‐tau, cholinesterase‐inhibitory, and synaptic‐modulating effects. Key medicinal species, including *Abelmoschus esculentus, Brassica oleracea, Cannabis sativa, Citrus reticulata, Lantana camara, Launaea taraxacifolia, Lawsonia inermis, Marrubium vulgare, Markhamia lutea, Persicaria minor, Pithecellobium dulce, Salvia aristata, Trigonella foenum-graecum*, and *Withania somnifera,* show significant cognitive and neuroprotective benefits in experimental AD models. Phytochemicals such as sulforaphane, nobiletin, trigonelline, diosgenin, verbascoside, withaferin A, and withanolides strongly modulate the amyloid, tau, oxidative, and inflammatory pathways. Clinical trials further support the therapeutic potential of several plant‐derived interventions for mild cognitive impairment and AD‐related dementia. Collectively, these findings highlight medicinal plants and their active constituents as compelling complementary or translational strategies for AD management, warranting further mechanistic and clinical validation. This review aims to evaluate the neuroprotective potential of medicinal plants and their bioactive compounds in preventing and managing AD by summarizing evidence from in vitro, in vivo, and clinical studies.

## 1. Introduction

Alzheimer’s disease (AD) is biologically characterized by extracellular β‐amyloid (Aβ) plaque deposition and intracellular neurofibrillary tangles composed of hyperphosphorylated tau, which together drive a cascade of progressive neurodegeneration. Clinically [1–3], AD manifests in both genetic and sporadic forms and typically presents with amnestic impairments, although several less common variants demonstrate prominent nonamnestic cognitive decline [4, 5]. A broad set of pathological disturbances accompany these hallmark lesions, including mitochondrial dysfunction, cerebral hypometabolism, oxidative stress, neuroinflammation, and disruption of the blood–brain barrier (BBB). Early structural damage typically follows the Papez circuit, which affects regions such as the hippocampal formation, entorhinal cortex, anterior thalamic nuclei, and posterior cingulate cortex. As AD progresses, atrophy extends across multiple cortical and subcortical areas, with the posterior cingulate cortex, precuneus, and medial orbitofrontal regions showing disproportionate vulnerability. In contrast, the auditory, sensorimotor, and premotor areas remain relatively preserved. Clinically, AD manifests as a gradual decline in memory, executive function, visuospatial processing, language, and speech production [6, 7]. These cognitive deficits impair daily activities and are often accompanied by behavioral and psychological symptoms of dementia (BPSD), which tend to emerge as the disease progresses [8]. Although the precise initiating mechanisms are still debated, two pathological signatures remain central to AD: extracellular Aβ fibrils forming senile plaques and intracellular neurofibrillary tangles composed of hyperphosphorylated tau. Current evidence suggests that Aβ accumulation represents an early and critical event in disease pathogenesis, with fibrillar deposits appearing years, often decades, before visible clinical symptoms [9]. The global health burden of AD continues to rise sharply. Data from the Global Burden of Disease Study place AD among the fastest‐growing causes of mortality. Projections estimate that the number of individuals living with AD will increase from 26.6 million in 2006 to approximately 107 million by 2050 [10], with approximately 16.5 million cases expected in Europe. Importantly, nearly 68% of this increase is anticipated in low‐ and middle‐income countries, reflecting demographic shifts and expanding life expectancy [11]. Most dementia cases occur in adults over 60 years of age. As the human lifespan continues to lengthen, the number of people affected by AD is growing rapidly, driving substantial research efforts to understand disease mechanisms and identify effective treatments. Despite these advances, current therapeutic strategies remain limited and do not halt or reverse the underlying neurodegenerative process [12]. Beyond its clinical and epidemiological burden, AD imposes a profound and escalating economic impact on healthcare systems and societies worldwide. Recent evidence indicates that the worldwide cost of dementia, largely driven by AD, has already exceeded US$1 trillion annually and is projected to rise dramatically with population aging and increased life expectancy [13]. A systematic economic analysis further suggests that global dementia‐related costs could reach approximately US$2 trillion by 2030, reflecting the growing demand for long‐term care, institutionalization, and informal caregiving. Moreover, projections based on the value‐of‐statistical‐life approach estimate that the total global economic burden of AD and related dementias was approximately US$2.8 trillion in 2019 and may increase substantially to as high as US$9 trillion by 2050, with a significant shift toward low‐ and middle‐income countries [13, 14]. These rising costs are driven not only by direct medical expenditures but also by indirect costs such as productivity loss and informal caregiving, which represent a substantial proportion of the total societal burden. Collectively, these projections underscore that AD is not only a major clinical challenge but also a critical economic issue, necessitating urgent investment in preventive strategies, early diagnosis, and more effective therapeutic interventions [13–15].

Currently approved therapies for AD target symptoms rather than the underlying neurodegenerative process. The U.S. FDA has endorsed acetylcholinesterase (AChE) inhibitors donepezil, galantamine, and rivastigmine and the N‐methyl‐D‐aspartate (NMDA) receptor antagonist memantine for clinical use, primarily to provide modest improvements in cognition and daily functioning. However, no single drug is capable of consistently controlling the broad spectrum of AD symptoms, making long‐term management challenging [16]. Existing medications largely focus on alleviating neurological and behavioral manifestations, yet they do not stop disease progression. Their use is further limited by side effects, modest therapeutic benefits, and reduced patient adherence, underscoring the urgent need for alternative therapeutic strategies [17]. Because of these limitations, increasing attention has focused on herbal and traditional medical systems, including Ayurveda and traditional Chinese medicine (TCM), which are valued for their natural origin, favorable safety profiles, and generally lower incidence of adverse effects than many modern pharmacological agents [18, 19]. In recent decades, the neuroprotective potential of natural products has been widely examined. Crude plant extracts, isolated phytochemicals, and standardized herbal formulations have shown meaningful promise in experimental models and early clinical evaluations for managing or delaying AD pathology [20, 21]. Many of these natural compounds demonstrate mechanisms relevant to AD, such as antioxidant effects, anti‐inflammatory actions, AChE inhibition, modulation of the amyloid and tau pathways, and protection against synaptic dysfunction, supporting their potential therapeutic role. Numerous phytochemicals with neuroactive properties continue to be evaluated for AD, adding to a growing body of evidence supporting their translational relevance [22]. Historically, many modern medicines have traced their origins to plant‐derived compounds, and large portions of early pharmacopeias were built around herbal preparations. While the rise of modern pharmacology has led to a substantial decline in the clinical use of traditional remedies, interest in herbal therapeutics has re‐emerged, particularly for chronic, psychiatric, and neurological disorders. Several factors contribute to this trend: dissatisfaction with the limited effectiveness of conventional drugs, the appeal of treatments aligned with cultural or personal beliefs, and a growing preference for greater autonomy in healthcare decisions [23]. Importantly, many plant‐derived constituents, including alkaloids, flavonoids, terpenoids, and diverse phenolic compounds, have demonstrated activity toward key brain receptors and enzymes. Many exhibit robust AChE inhibitory activity, suggesting that herbal medicines may hold meaningful value in neurological disease management [24]. Both preclinical and clinical studies further support the scientific basis for the use of medicinal plants for treating cognitive disorders, reinforcing their potential as complementary or alternative options for AD therapy [23]. The aim of this study is to comprehensively evaluate the therapeutic potential of medicinal plants and their bioactive phytoconstituents for the prevention and management of AD. It examines key neuroprotective mechanisms, including antioxidant and anti‐inflammatory effects, AChE inhibition, and modulation of amyloid‐β (Aβ) and tau pathology, based on evidence from in vitro, in vivo, and clinical studies. The study integrates findings on whole plant extracts, isolated phytochemicals, and dietary applications to provide a coherent perspective on their translational potential for mitigating AD‐related cognitive decline.

## 2. Methodology

### 2.1. Literature Search Strategy

A comprehensive and semisystematic literature search was conducted using major scientific databases, including Google Scholar, PubMed, Cochrane Library, ScienceDirect, and Scopus. The search strategy employed Boolean combinations of keywords to maximize retrieval of relevant studies, including “Alzheimer’s disease” OR “dementia” AND (“medicinal plant” OR “plant extract” OR “phytochemicals”) AND (“memory enhancement” OR “cognitive improvement” OR “neuroprotective”) AND (“in vitro” OR “in vivo” OR “preclinical” OR “clinical trials”). In addition, targeted searches were performed using the scientific names of frequently studied medicinal plants (e.g., *Withania somnifera*, *Abelmoschus esculentus*, *Brassica oleracea*) combined with terms such as “isolated compound” or “phytochemical” to ensure inclusion of studies investigating both crude extracts and purified bioactive constituents.

To address the diversity of available medicinal plants, a semisystematic selection strategy was adopted. Studies were prioritized based on (i) the strength and consistency of experimental evidence (including reproducibility across in vitro, in vivo, and clinical studies); (ii) the presence of clearly identified bioactive compounds with defined mechanisms of action; and (iii) relevance to key pathological features of AD, such as Aβ aggregation, tau hyperphosphorylation, oxidative stress, cholinergic dysfunction, and neuroinflammation. Preference was given to medicinal plants and phytochemicals that demonstrated multitarget neuroprotective effects, as these are considered more therapeutically relevant in complex neurodegenerative disorders like AD.

The review included studies published between 2015 and 2026, yielding a total of 174 relevant articles. Among these, 50 studies were published between 2015 and 2020, while 124 studies were published between 2021 and 2026, reflecting the growing research interest in plant‐based neuroprotective strategies.

### 2.2. Inclusion and Exclusion Criteria

The inclusion criteria encompassed (i) in vitro studies; (ii) in vivo preclinical studies using validated animal models of AD or cognitive impairment; and (iii) clinical trials assessing cognitive or neurological outcomes. Studies were required to evaluate plant extracts or isolated phytochemicals with demonstrated effects on memory enhancement, antioxidant activity, cholinesterase inhibition, antineuroinflammatory responses, or other mechanisms directly relevant to AD pathology. Data extracted from eligible studies included publication year, plant species, plant part used, type of extract or isolated compound, experimental model, dosage, treatment duration, observed pharmacological effects, and mechanistic insights.

Studies were excluded if they (i) did not specifically investigate AD or cognitive impairment; (ii) lacked mechanistic or efficacy‐related data; (iii) were review articles, editorials, or conference abstracts without primary data; or (iv) were not published in English.

### 2.3. Study Scope

**FIGURE 1:**
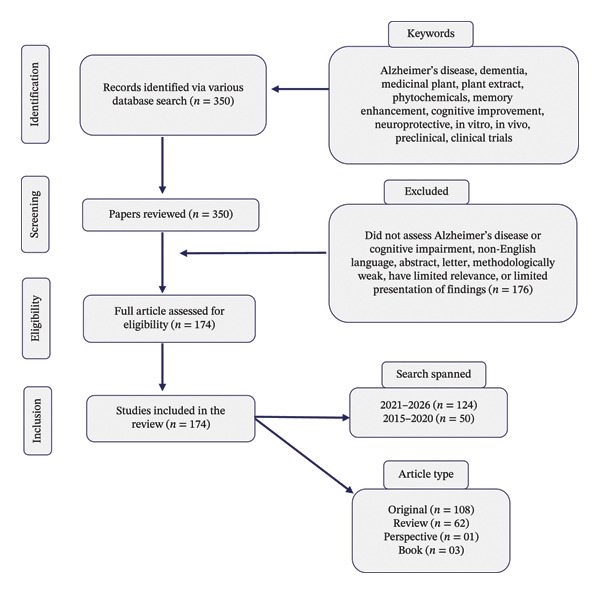
Flow diagram illustrating the review process for studies on Alzheimer’s disease. This figure illustrates the four stages of the systematic review process: identification, screening, eligibility, and inclusion. The diagram also provides an overview of the search periods and the categorization of article types, including original research, reviews.

Given the extensive number of medicinal plants reported in the literature, it was not feasible to include all species with potential neuroprotective effects. Therefore, this review selectively focused on representative plants and phytochemicals with substantial and well‐characterized evidence, particularly those supported by multiple independent studies or demonstrating translational relevance. This selective approach ensures clarity, avoids overgeneralization, and highlights the most promising candidates for future therapeutic development. Overall, this semisystematic methodology provides a transparent and reproducible framework for study selection while maintaining a critical focus on high‐quality and mechanistically relevant evidence (Figure 1).

## 3. Therapeutic Applications

### 3.1. Medicinal Plant and Their Benefits for AD

**FIGURE 2:**
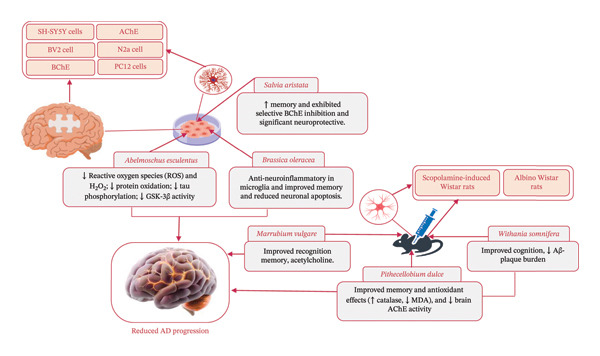
Proposed in vivo and in vitro mechanisms of action of medicinal plant in Alzheimer.

The medicinal plants included in this review were selected based on consistent experimental evidence from AD relevant models, including in vitro enzymatic assays, neuronal cell‐based studies, and in vivo models of cognitive impairment and neuroinflammation. Priority was given to species demonstrating multitarget neuroprotective mechanisms such as antioxidant activity, anti‐amyloid effects, cholinesterase inhibition, and modulation of neuroinflammatory signaling pathways. Accordingly, Table 1 compiles 14 well‐studied medicinal plants with consistent pharmacological evidence supporting their anti‐AD potential. Among these, *A. esculentus* Linn. (okra) demonstrates strong neuroprotective activity by reducing oxidative stress, tau hyperphosphorylation, GSK‐3β activity, and Aβ‐induced neuronal apoptosis, while also improving cognitive outcomes through antioxidant and anti‐inflammatory mechanisms in both cellular and animal models. Similarly, *B. oleracea* L. exhibits antineuroinflammatory effects in microglia and improves memory performance in scopolamine‐induced cognitive impairment models, indicating its role in neuroprotection and neuronal survival. *Cannabis sativa* L. seeds have been reported to enhance learning and memory while reducing hippocampal inflammatory cytokines and neuronal damage in LPS‐induced neuroinflammation and aging models. In parallel, *Citrus reticulata* Blanco shows dose‐dependent neuroprotective effects, including reduction of hippocampal neuronal degeneration and significant AChE inhibition, contributing to improved cognitive performance. *Lantana camara* L. demonstrates both behavioral and molecular neuroprotection by improving memory and suppressing proinflammatory gene expression (IL‐1β, IL‐6, TNF‐α, and COX‐2), while its essential oil also exhibits strong AChE inhibitory and antioxidant properties. Likewise, *Launaea taraxacifolia* (Willd.) provides robust antioxidant defense by restoring glutathione levels, reducing lipid peroxidation, and improving neurobehavioral outcomes across multiple neurotoxicity models. *Lawsonia inermis* L. shows broad neuroprotective potential through improvement of learning and memory, suppression of anxiety and motor dysfunction, and enhancement of cholinergic function via increased acetylcholine (ACh) levels and reduced AChE activity. In addition, *Marrubium vulgare* L. supports cholinergic preservation and neurotrophic signaling by enhancing hippocampal ACh, brain‐derived neurotrophic factor (BDNF), and reducing oxidative stress, while also inhibiting AChE and butyrylcholinesterase (BChE) enzymes. *Markhamia lutea* (Benth.) contributes to anti‐AD activity through inhibition of AChE, BChE, and Aβ aggregation along with antioxidant effects. Similarly, *Persicaria minor* Opiz enhances memory and hippocampal neurotransmission (ACh and GABA modulation), while activating neuroprotective signaling pathways such as Nrf2/ARE and suppressing NF‐κB‐mediated inflammation. *Pithecellobium dulce* Benth. shows consistent anti‐amnesic effects by improving memory, reducing oxidative stress, and inhibiting brain AChE activity across multiple experimental models. *Salvia aristata* Aucher ex Benth. further supports neuroprotection through antioxidant, metal‐chelating, and selective cholinesterase inhibitory effects, alongside improvements in memory performance. *Trigonella foenum-graecum* L. (fenugreek) enhances learning and memory while preventing neuronal loss, supporting its role in cognitive preservation. Finally, *W. somnifera* (L.) Dunal (ashwagandha) demonstrates one of the most comprehensive anti‐AD profiles, including reduction of Aβ plaques, neuroinflammation, oxidative stress, and improvement of neuronal calcium homeostasis and cognitive performance (Table 1) (Figure 2).

**TABLE 1:** Medicinal plants beneficial for AD.

Plant name	Family	Part	Extraction	Study type	Dose	Pharmacological activity	Ref
*Abelmoschus esculentus* Linn.	Malvaceae	Okra fruits	Ethanol	In vitro, human neuroblastoma SH‐SY5Y cells		↓ Reactive oxygen species (ROS) and H_2_O_2_, ↓ protein oxidation, ↓ tau phosphorylation (Ser199, Ser202, Ser396), ↓ GSK‐3β activity, ↓ intracellular iron suggesting protection against AD‐linked oxidative stress and tau pathology	[25]
				In vitro, Aβ‐induced neuronal apoptosis models (cellular assays)	5, 25 μg/mL	Prevent Aβ‐induced neuronal damage by modulating DPP‐4 and insulin‐resistance pathways, suggesting AE as a potential adjuvant for protecting against Aβ‐related neurodegeneration.	[26]
		Okra fruits	Aqueous, ethanol	In vivo, Aβ_1-42_ intracerebroventricular mouse model	100 and 300 mg/kg	↓ neuroinflammation, ↑ antioxidant effect	[27]
		Seed	Ethanol	In vitro, acetylcholinesterase (AChE) inhibition assays	10 and 20 μg mL^−1^	Exhibits potent AChE inhibition, robust antioxidant activity, and significant behavioral improvement.	[28]
				In vivo, AlCl_3_‐induced Wistar albino rats	500 mg/kg		

*Brassica oleracea* L.	Brassicaceae		Methanol	In vivo, scopolamine‐induced memory impairment in mice.	100 and 200 mg/kg	Antineuroinflammatory in microglia and improved memory and ↓neuronal apoptosis	[29]
				In vitro, murine microglia cell (BV2), neuroblastoma cell (N2a)			

*Cannabis sativa* L.	Cannabaceae	Seed		In vivo, LPS‐induced neuroinflammation	1 g/kg	↑ learning and memory, ↓ hippocampal proinflammatory cytokines and attenuated hippocampal neuronal damage.	[30]
		Seed	Aqueous	In vivo, D‐galactose‐induced aging/cognitive impairment in rats	200 and 400 mg/kg	↑ learning and memory	[31]

*Citrus reticulata* Blanco	Rutaceae	Peel		In vivo, Wistar rats, trimethyltin‐induced hippocampal damage	56.25, 112.5, 225 mg/kg/day	↓ number of degenerating hippocampal neurons	[32]
		Leaves	Methanol	In vivo, scopolamine‐induced amnesia model	200 and 400 mg/kg	Significant AChE inhibitory and antioxidant activity ↑ memory, ↓ brain AChE activity	[33]

*Lantana camara* L.	Verbenaceae	Leave	Hydroethanolic	In vivo, scopolamine‐induced memory impairment in zebrafish and mice	10, 30, 100 mg/kg	↑ memory and suppressed neuroinflammatory gene expression (IL‐1β, IL‐6, TNF‐α, COX‐2) in hippocampus/cortex.	[34]
		Flower	Essential oil by hydrodistillation	In vitro acetylcholinesterase (AChE) inhibition assays		AChE inhibition and antioxidant effect	[35]

*Launaea taraxacifolia* (Willd.) Amin ex C. Jeffrey	Asteraceae	Leaves	Aqueous	In vivo, cisplatin‐induced neurotoxicity in Wistar rats	100 and 400 mg/kg	Restored brain antioxidant status (↑GSH), ↓ lipid peroxidation (↓MDA), ↓ neuronal cell death, antioxidant, neuroprotective activity	[36]
		Leaves		In vivo, Wistar rats	100 and 200 mg/kg	↓ oxidative stress, and improved neurobehavioural, antioxidant, anti‐inflammatory neuroprotection effect	[37]
		Leaves	Ethanol	In vivo, aluminum chloride (AlCl_3_) induced neurotoxicity in Wistar rats	274–822 mg/kg	↑ motor coordination, cognitive flexibility, and visuospatial function.	[38]

*Lawsonia inermis* L.	Lythraceae	Leaves and flowers	Aqueous	In vivo, zebrafish AD model (sodium‐valproate induced)	15, 30, 250, 500 μg	↓ anxiety, degeneration, and motor dysfunction with a significant ↑ memory and learning.	[39]
		Leaves	Ethanol	In vivo	25 mg/kg	↑ learning, memory, ↑ antioxidant effect	[40]
		Seeds	Methanol	In vivo, D‐galactose induced AD in Wistar rats	150 mg/kg	↑ ACh and ↓ AChE activity in the cerebral cortex of AD rats	[41]

*Marrubium vulgare* L.	Lamiaceae	Aerial	Water	In vivo, Wistar rats, scopolamine induced memory impairment	200 mg/kg	↑ recognition memory, hippocampal ACh levels; ↑ brain noradrenaline; restored *p*‐CREB and upregulated BDNF and Bcl‐2; ↓ oxidative stress markers suggests cholinergic preservation, neurotrophic	[42]
		Aerial	Hydroethanolic	In vitro, AChE, butyrylcholinesterase (BChE) assays	100–1000 μg/mL	AChE, BChE, and tyrosinase were significantly inhibited.	[43]
		Whole aerial plant	Aqueous/methanol	In vivo, mice, scopolamine‐induced dementia model	700 and 1400 mg/kg	↑ memory by modulating glutamatergic activity and ↓ oxidative stress	[44]

*Markhamia lutea* (Benth.) K. Schum	Bignoniaceae	Leaves and flowers	Ethanol	In vitro, AChE, BChE assays		↑ Antioxidants, Inhibit AChE, BChE and Aβ‐amyloid	[45]

*Persicaria minor* Opiz	Polygonaceae	Leaf	Aqueous	In vivo	200 and 300 mg/kg	↑ recognition and spatial memory, ↑ hippocampal ACh and GABA	[46]
		Leaf	Ethanolic	In vitro, SH‐SY5Y human neuroblastoma cells	0.5–1000 μg/mL	Neuroprotection, activation of Nrf2/ARE, modulation of NF‐κB/IκB and MAPK signaling; ↑ ACh levels	[47]

*Pithecellobium dulce* benth.	Fabaceae	Leaves	Ethanolic	In vivo, scopolamine‐induced memory impairment in rats	200 and 400 mg/kg	↑ memory and locomotor measures, antioxidant effects (↑ catalase, ↓ MDA), and ↓brain AChE activity	[48]
		Aerial parts	Methanol	In vivo, aluminum chloride (AlCl_3_)‐induced cognitive dysfunction in zebrafish and mice	200 and 400 mg/kg	↑ memory, inhibited elevated AChE activity induced by AlCl_3_, and ↓oxidative stress	[49]
		Leaves	Methanol	In vitro, AChE inhibition	19.23 μg/mL	Antioxidant activity, acetylcholinesterase, dopamine, and noradrenaline inhibition activities and ↑ ACh level in the brain.	[50]
				in vivo, scopolamine‐induced Alzheimer’s model in rats	100, 250, and 500 mg/kg		

*Salvia aristata* Aucher ex Benth.	Lamiaceae	Aerial parts	Hydroalcoholic and dichloromethane	In vitro, PC12 cells	2.5, 5, 10, and 20 μg/mL	Significant neuroprotective, antioxidant, and metal‐chelating properties, memory improvement.	[51]
				in vivo, scopolamine‐induced memory impairment in albino Wistar rats	400 mg/kg		
		Aerial parts	Hydrodistillation (essential oil)	In vitro, PC12 cells; AChE, BChE assays	63.5, 125, 250, and 500 μg/mL	Exhibited selective BChE inhibition and significant neuroprotective effects	[52]

*Trigonella foenum-graecum* L.	Fabaceae	Seed		In vivo	1.0 g/kg	↑ learning and memory, and prevented neuronal loss	[53]
		Seed	Methanol	In vivo, scopolamine‐induced amnesia in mice	200 mg/kg	Improved acquisition and retention in behavioral memory	[54]

*Withania somnifera* (L.) Dunal	Solanaceae	Root	Aqueous	In vivo, thioacetamide (TAA)‐induced hepatic encephalopathy rat model	200 and 400 mg/kg	Improved locomotor and cognitive deficits, ↑ recognition index and spontaneous alternation (%), reduced brain MDA and iNOS, increased GSH, Nrf2 and HO‐1, and downregulated NF‐κB/MAPK signaling	[55]
		Root	Methanol	In vivo	200 and 400 mg/kg	Improved cognition, ↓ Aβ‐plaque, and ↑ NCX3 expression (a Ca^2+^ exchanger implicated in neuronal Ca^2+^ handling), and ↓ neuroinflammation	[56, 57]

#### 3.1.1. *A. esculentus* (L.) Moench

*A. esculentus*, commonly known as okra, is a member of the Malvaceae family and is widely cultivated across Africa, South Asia, and the Middle East. Traditional medical systems describe its use for managing diabetes, digestive disturbances, and inflammation and as a general restorative tonic, reflecting its long history as both a food and therapeutic plant [58]. Phytochemical investigations have shown that okra fruits, seeds, and mucilage contain abundant polysaccharide, flavonoid, phenolic, vitamin, and unsaturated fatty acid components linked to antioxidant, anti‐inflammatory, hypoglycemic, and lipid‐modulating effects [59]. Emerging evidence indicates that okra polysaccharides exert prebiotic effects that modulate gut microbiota composition and metabolic homeostasis, thereby influencing the gut–brain axis and contributing to neuroprotection in AD models [60]. These bioactive properties provide a strong rationale for evaluating okra in neurodegenerative disorders such as AD, where oxidative stress, neuroinflammation, and metabolic dysfunction, including brain insulin resistance, play central roles in disease progression [27, 60]. Notably, dietary polysaccharides, including those from okra, have been shown to regulate AD‐related pathology through microbiota–gut–brain axis signaling and metabolic pathways, highlighting their multitarget therapeutic relevance [61]. Emerging preclinical evidence supports the neuroprotective potential of *A. esculentus*. This neuroprotective potential is increasingly linked to metabolic modulation, as okra polysaccharides have been shown to reverse high‐fat diet‐induced metabolic dysfunction and cognitive impairment via insulin signaling pathways closely associated with gut–brain axis communication [62]. Flavonoid‐rich fractions and polysaccharide extracts have shown cognitive benefits in AD models, improving performance in the Y‐maze and Morri’s water maze, reducing Aβ_1_–_42_‐induced memory impairment, and attenuating oxidative and inflammatory markers in rodent and transgenic APP systems [27]. A recent study demonstrated that okra seed extract exhibits both in vitro and in vivo anti‐AD activity, including the inhibition of key pathological enzymes and protection against neuronal injury, highlighting its promise as a source of lead compounds for drug development [28]. Mechanistic studies using human neuroblastoma SH‐SY5Y cells revealed that ethanol extracts of okra fruit reduce reactive oxygen species, suppress protein oxidation, decrease tau phosphorylation at Ser199, Ser202, and Ser396, inhibit GSK‐3β activity, and lower intracellular iron, collectively suggesting protection against AD‐linked oxidative and tau‐related pathology [25]. Additional cellular assays revealed that low concentrations of okra extract (5 and 25 μg/mL) prevent Aβ‐induced neuronal apoptosis by modulating DPP‐4 and insulin resistance pathways, supporting its role as an adjuvant in insulin‐related neurodegenerative processes [26]. In vivo, aqueous and ethanol fruit extracts (100–300 mg/kg) counteract Aβ_1_–_42_‐induced cognitive deficits, reduce neuroinflammation (lower TNF‐α and IL‐1β), improve antioxidant systems, restore BDNF levels, and activate the CREB/ERK and PI3K/AKT/GSK‐3β signaling cascades, indicating broad neuroprotective effects [27]. Recent mechanistic studies further demonstrate that okra seed polysaccharides ameliorate neuroinflammation and cognitive decline by regulating multiple metabolic and signaling axes, including AMPK/SIRT1 and PI3K/AKT pathways, which are functionally linked to gut microbiota‐derived metabolic regulation [63]. Okra seed extracts also demonstrate strong AChE inhibitory activity in vitro, accompanied by antioxidant effects and improvements in neurotransmitter balance, oxidative markers, and histopathology in AlCl_3_‐treated rats, further supporting their anti‐Alzheimer’s potential [28]. The pharmaceutical applications of okra highlight several translational opportunities. Importantly, these applications should be understood within the framework of gut–brain axis modulation, as okra‐derived polysaccharides act as prebiotic substrates that enhance beneficial microbial populations and short‐chain fatty acid production, thereby influencing neuroinflammation and cognitive function in AD [60]. Okra polysaccharides can be developed as oral nutraceuticals to target insulin signaling and modulate the gut–brain axis, offering a promising approach for treating metabolic and neurodegenerative disorders. Flavonoid‐enriched fractions from okra may serve as neuroprotective phytopharmaceuticals capable of reducing oxidative stress and Aβ toxicity, thereby mitigating AD‐related pathology. Additionally, seed oil and peptide isolates have potential as adjuvants that enhance neuronal membrane integrity and synaptic plasticity, contributing to improved cognitive function. The natural mucilage of okra can be utilized in mucoadhesive formulations for controlled‐release oral or buccal delivery of neuroactive extracts, ensuring sustained bioavailability. Furthermore, prebiotic microcapsules that combine okra polysaccharides with probiotic strains effectively modulate microbiota‐driven neuroinflammation, supporting the gut–brain axis and overall neuronal health [59, 60].

#### 3.1.2. *B. oleracea* L.

*B. oleracea* (family Brassicaceae) is a highly polymorphic biennial herb encompassing economically and nutritionally important cultivars such as cabbage, broccoli, cauliflower, and kale, characterized botanically by thick succulent stems, glabrous bluish‐green leaves with a waxy cuticle, and compact inflorescences forming dense heads or curds depending on the cultivar; the plant typically exhibits a cruciform flower structure with four yellow petals and siliqua‐type fruits. Native to the Mediterranean region, it is now widely cultivated across Europe, Asia, and tropical regions due to its adaptability to diverse agro‐climatic conditions, with significant production in temperate and subtropical zones. *B. oleracea* has long been used as both a dietary vegetable and a medicinal plant for managing inflammatory disorders, gastrointestinal disturbances, and metabolic conditions, reflecting its rich nutritional profile and bioactive phytochemical content. The plant is particularly valued for its glucosinolates (e.g., glucoraphanin), which upon enzymatic hydrolysis yield isothiocyanates such as sulforaphane, along with flavonoids, phenolic acids, vitamins (C, E), carotenoids, and essential minerals, collectively contributing to antioxidant, anti‐inflammatory, and chemoprotective effects [64]. From a neuropharmacological perspective, these phytoconstituents especially sulforaphane have attracted considerable attention in AD research due to their multitarget actions, including modulation of oxidative stress, neuroinflammation, Aβ aggregation, and tau pathology; mechanistically, sulforaphane activates the Nrf2 signaling pathway, enhancing endogenous antioxidant defenses and reducing neurodegenerative cascades [65]. Experimental evidence supports these claims, as *B. oleracea* methanolic extracts demonstrated significant cognitive improvement and reduction of neuronal apoptosis in scopolamine‐induced memory‐impaired mice at doses of 100 and 200 mg/kg, alongside suppression of microglial‐mediated neuroinflammation (Table 1), indicating a broad neuroprotective profile. In vitro investigations using BV2 microglial and N2a neuroblastoma cells further confirm anti‐inflammatory and neuroprotective effects, aligning with observed inhibition of β‐secretase (BACE1) activity by sulforaphane at micromolar concentrations (0.03–3.0 μM), suggesting a direct role in attenuating amyloidogenic processing pathways (Table 1). In vivo studies extend these findings, where sulforaphane administration (1 mg/kg) reduced cognitive decline and AD‐like neuropathology via Nrf2 activation, particularly relevant in comorbid conditions such as Type 2 diabetes mellitus, highlighting its systemic neuroprotective potential (Table 2). Moreover, transgenic PS1V97L mouse models treated with sulforaphane (5 mg/kg) exhibited decreased tau phosphorylation, oxidative stress, and inflammatory markers, accompanied by improved cognitive performance (Table 2), reinforcing its disease‐modifying potential. Additional mechanistic studies reveal that sulforaphane can reduce Aβ and tau accumulation by enhancing proteostasis pathways, including upregulation of heat shock proteins and CHIP‐mediated clearance systems, further substantiating its role in targeting core AD pathology [83]. Despite these promising findings, critical evaluation indicates that most evidence remains preclinical, with limited human clinical validation, and issues such as bioavailability, variability in phytochemical content across cultivars, and dose standardization remain significant challenges for pharmaceutical translation. Furthermore, while sulforaphane exhibits good BBB penetration and pleiotropic effects, its stability and rapid metabolism may limit sustained therapeutic efficacy, necessitating formulation optimization and controlled clinical trials. Nevertheless, the convergence of ethnobotanical relevance, rich phytochemistry, and robust experimental evidence positions *B*. *oleracea* as a promising candidate for AD prevention and adjunct therapy, although rigorous clinical validation and pharmacokinetic standardization are essential before its integration into evidence‐based neurotherapeutics.

**TABLE 2:** Pharmacological effects of the active phytoconstituents of selected medicinal plants against AD.

Active compound	Source	Study type	Dose	Pharmacological activity	Ref
Sulforaphane (**1**)	*Brassica oleracea* L. (Brassicaceae)	In vitro, BACE1 activity	0.03, 0.3, 1.5, and 3.0 µM	Novel BACE1 inhibitor with high potency and selectivity and good BBB penetration property	[66]
		In vivo	1 mg/kg	↓ cognitive decline and AD‐like lesions via Nrf2 activation, suggesting benefit for T2DM‐related cognitive impairment.	[67]
		In vivo, PS1V97L transgenic (Tg) mice	5 mg/kg	↓ tau phosphorylation, oxidative stress, and inflammation, and ↑ cognition	[68]

Cannabigerol (**2**)	*Cannabis sativa* L. (Cannabaceae)	In vitro, NSC‐34 cells	1, 2.5, 5, 7.5, 10, 12.5, 15 and 20 µM	↓ microglial activation, ↓ inflammatory mediators/oxidative stress, and shows neuroprotective potential.	[69]
		In vivo	5–10 mg/kg		
Tetrahydrocannabinol (**3**)		In vivo, 5xFAD tg‐mice	0.002 mg/kg	↓ cognition, ↑ amyloid/tau changes and oxidative stress, and may interact with Aβ while modulating AChE and neuroinflammation.	[70]

Nobiletin (**4**)	*Citrus reticulata* Blanco (peel) (Rutaceae)	In vivo	50 mg/kg	↑ motor and memory performance; ↓ AChE activity, ↓Aβ and BACE1, ↓ neuroinflammation/oxidative stress, ↑autophagy via SIRT1/FoxO3a	[71]
Tangeretin (**5**)		In vivo, global cerebral ischemia (BCCAO) in rats	5, 10, and 20 mg/kg	↑ cognition and memory by ↑ Ach levels through the amelioration of AchE enzyme activity	[72]

β‐Sitosterol (**6**), 3‐O‐β‐acetyloleanolic acid (**7**), 3‐O‐(Z)‐coumaroyl oleanolic acid (**8**), betulinic acid (**9**), and oleanolic acid (**10**)	*Lawsonia inermis* L. (Lythraceae)	In vitro, AChE/BChE inhibition assay	1, 10, 20, 40 μg/mL	Shows potent, selective BChE inhibition, with compounds 2 and 5 being the strongest inhibitors.	[73]

Trigonelline (**11**)	*Trigonella foenum-graecum* L. (Fabaceae)	In vivo, transgenic AD model (5XFAD mice)	10 mg/kg	↑ object‐recognition and location memory, reverses axonal degeneration, crosses the brain, and targets creatine kinase B.	[74]
Diosgenin (**12**)		In vivo, transgenic AD model (5XFAD mice)	1–10 mg/kg	↑ axonal growth and regeneration, restores memory function, ↓ axonal atrophy, ↑ memory and cognitive function	[75]

Polygonumins B and C (**13,14**)	*Persicaria minor* Opiz (Polygonaceae)	In vitro, enzyme assays AChE and BChE	100 μg/mL	Strong AChE inhibition and potent antioxidant activity.	[76]

Afzelin (**15**)	*Ribes fasciculatum* var. chinense MAX. (Grossulariaceae)	In vivo, scopolamine induced in C57BL/6 mice	100 ng/μL	↑ cognitive and memory function	[77]

Verbascoside (**16**)	*Verbascum sinuatum* L. (Scrophulariaceae)	In vitro, BV2 and N2a cells	50 and 100 μM	Prevents microglia and astrocyte activation by blocking NF‐κB‐p65 nuclear translocation, suppresses proinflammatory mediators, boosts anti‐inflammatory signals, and protects neuroinflammation	[78]
		In vivo, B6C3‐Tg APP/PS1 mice	10 mg/kg		
		In vivo	30, 60 and 120 mg/kg	↑ learning and memory	[79]
		In vitro, U251 cells	0.25 and 1 μM	↑ mitochondrial and ER morphology, ↑ memory and cognition, inhibits apoptosis, and ↓ Aβ deposition, tau tangles, 4‐HNE, and MANF expression.	[80]
		In vivo, APP/PS1 transgenic mice	10 mg/kg		

Withaferin A (**17**)	*Withania somnifera* (L.) Dunal (Solanaceae)	In vitro, human neuroblastoma cell	0.5–2 μM	↓ secreted Aβ_40_ levels and rescued Aβ‐induced neurotoxicity. ↓ Aβ40; ↑ neuronal morphology, anti‐Aβ neuroprotective agent.	[81]
Withanolide A (**18**), withanolide B (**19**), withanoside IV (**20**) and withanoside V (**21**)		In vitro, anti‐Aβ aggregation and cytoprotection assays on neuronal cell lines	10, 20, 40, 60, 80, 100 μM	Inhibited Aβ_1-42_ aggregation, ↓ Aβ‐induced cytotoxicity and oxidative stress. ↓ ROS and ↑ cell survival in Aβ‐treated cultures.	[82]

#### 3.1.3. *C. sativa* L.

*C. sativa,* commonly known as cannabis, hemp, or marijuana, is a chemically diverse member of the Cannabaceae family with long‐standing cultural, nutritional, and medicinal relevance. Across Africa, Asia, Europe, and the Middle East, its leaves, seeds, and inflorescences have been traditionally used for pain, inflammation, gastrointestinal complaints, mood disturbances, and memory‐related symptom patterns, as consistently documented in ethnobotanical reports [84, 85]. Modern phytochemical research has confirmed that *C. sativa* contains a broad spectrum of bioactive constituents, particularly phytocannabinoids such as tetrahydrocannabinol (THC) and cannabidiol (CBD), alongside numerous terpenes, flavonoids, and nutrient‐rich seed oils. This chemical complexity provides a strong biological basis for both historical use and increasing pharmaceutical interest [85]. Contemporary therapeutic development has shifted from traditional preparations to standardized formulations. CBD has progressed to clinical application for epilepsy and is under active investigation for anxiety, psychosis, and chronic pain, whereas purified or synthetic cannabinoids are being evaluated for spasticity, persistent pain, and palliative care. These advances have stimulated innovations in formulation science, including oil extracts, transdermal systems, and inhalation products, and established regulatory pathways for cannabinoid‐based medicines [85, 86]. The multifaceted neuroprotective potential of cannabis‐derived compounds in AD. CBD has been shown to have anti‐inflammatory, antioxidant, and antiamyloid effects in cellular and animal AD models, where it modulates microglial activation, reduces proinflammatory cytokine release, and influences signaling pathways related to synaptic plasticity and neurogenesis [87, 88]. While these findings suggest pleiotropic potential, some effects are inferred from independent studies rather than demonstrated concurrently. Studies have identified multiple molecular targets for CBD beyond CB1/CB2, including the PPARγ, TRPV1 and serotonergic pathways, which helps explain diverse neuroprotective signals across models and suggests rational bases for combination formulations that include nonpsychoactive cannabinoids and supportive terpenes [89]. Low‐dose THC and combined THC/CBD preparations reduce the β‐amyloid burden and improve specific memory outcomes in transgenic mice, suggesting the potential for both symptomatic relief, such as agitation, appetite changes, and sleep disturbances, and disease‐modifying effects through interactions with the endocannabinoid system and additional receptor targets [87, 88]. Recent preclinical studies reinforce these observations. *C. sativa* seed preparations prevent LPS‐induced learning and memory deficits in vivo; reduce hippocampal IL‐1β, IL‐6, and TNF‐α levels; and protect neuronal integrity, demonstrating clear antineuroinflammatory activity [30]. Aqueous seed extracts also improve learning and memory in D‐galactose‐induced aging models (200–400 mg/kg), increasing SOD activity, lowering lipid peroxidation, and improving hippocampal histology findings aligned with antioxidant‐driven neuroprotection [31]. Cannabigerol (CBG) reduces microglial activation and oxidative stress at micromolar concentrations (1–20 μM) in NSC‐34 cells and shows in vivo neuroprotective potential through anti‐inflammatory and receptor‐modulating mechanisms involving PPARγ and TRP channels [69]. Ultralow‐dose THC (0.002 mg/kg) has been reported to improve cognition in 5xFAD transgenic mice, with reductions in amyloid/tau pathology and oxidative stress, possibly through direct interactions with Aβ and modulation of AChE and inflammatory pathways [70].

#### 3.1.4. *C. reticulata* Blanco

*C. reticulata* (mandarin or tangerine), a member of the Rutaceae family, has a long history of traditional medicinal use throughout East Asia and Southeast Asia. Dried peel preparations (commonly known as *chenpi*) as well as fresh fruit have been widely used to alleviate dyspepsia, cough, excessive phlegm, hiccups, and general gastrointestinal discomfort, as consistently reported in ethnopharmacological surveys and traditional medicine texts [90]. Phytochemical analyses revealed that *C. reticulata* is enriched with flavanone glycosides and polymethoxylated flavones, including hesperidin, narirutin/naringin, nobiletin, and tangeretin, in addition to terpene‐rich essential oils dominated by limonene. These constituents are concentrated primarily in the peel and seeds and are responsible for the strong antioxidant, anti‐inflammatory, and neuroactive profiles [91, 92]. These reported mechanisms are based on individual experimental studies; however, their concurrent multitarget effects have not always been validated within a single AD‐relevant model. Because mandarin peel is generated in large quantities as an agrofood byproduct, current pharmaceutical and nutraceutical research has focused on sustainable extraction and valorization strategies. Techniques such as pulsed electric field processing and ultrasound‐assisted extraction have improved compound yield and purity, enabling the conversion of mandarin peel waste into standardized extracts and enriched fractions suitable for functional foods, oral supplements, and topical formulations. This circular approach supports both the environmental sustainability and the development of phytochemical‐based health products [93, 94]. Hesperidin and naringin exhibit potent antioxidant and anti‐inflammatory effects, modulate signaling pathways associated with neuronal survival, reduce AChE activity, and enhance behavioral performance in rodent memory models. Polymethoxylated flavones such as nobiletin and tangeretin provide complementary neuroprotective actions, including attenuation of amyloid and tau pathology, preservation of synaptic integrity, and improvement of learning and memory across amyloid‐, scopolamine‐, and ischemia‐based systems [95]. These compounds act through pleiotropic mechanisms, including suppression of reactive oxygen species, downregulation of neuroinflammatory mediators, interference with Aβ aggregation and BACE1 activity, support of autophagy, and enhancement of cholinergic neurotransmission. This multifaceted activity has positioned *C. reticulata* constituents as promising candidates for neuroprotective nutraceuticals and early‐stage AD drug discovery [95, 96]. Recent in vivo studies support these mechanistic insights. Mandarin peel extracts significantly reduce hippocampal neuronal degeneration in trimethyltin‐induced neurotoxicity models (56.25–225 mg/kg/day) [32]. Methanolic leaf extracts improved scopolamine‐induced memory impairment at 200–400 mg/kg, demonstrating strong AChE inhibitory and antioxidant effects, with reductions in brain lipid peroxidation and AChE activity [33]. Nobiletin (50 mg/kg/day) improved motor and memory performance while lowering AChE, Aβ, and BACE1 levels, reducing neuroinflammation and oxidative stress, and activating SIRT1/FoxO3a‐dependent autophagy pathways in vivo [71]. Tangeretin, administered at 5–20 mg/kg, enhances cognition in ischemia‐induced injury models by increasing ACh availability through the amelioration of AChE activity [72].

#### 3.1.5. *L. camara* L.

*L. camara* (Verbenaceae) is a perennial, aromatic, woody shrub widely recognized for its ethnomedicinal relevance and emerging neuropharmacological potential, particularly in neurodegenerative disorders such as AD. Botanically, it is characterized by a multibranched thorny habit reaching 0.5–3 m in height, with opposite, serrated leaves that are ovate to oblong and rough in texture due to trichomes. Its inflorescences are compact, umbel‐like heads bearing multicolored florets (yellow, orange, pink, red, or white), which often change color with maturation, and it produces small berry‐like drupes that turn dark purple when ripe. The plant exhibits high morphological variability due to hybridization and environmental adaptability, making it a widely distributed invasive species [97]. Geographically, *L. camara* is native to tropical America but has naturalized across Asia, Africa, Australia [98] and Oceania, thriving in disturbed habitats, forest margins, and agricultural lands, particularly in tropical and subtropical climates [99]. Ethnobotanically, it is deeply integrated into traditional medicinal systems such as Ayurveda, where leaf decoctions, poultices, and extracts are used for treating fever, skin infections, respiratory disorders, rheumatism, and gastrointestinal ailments, reflecting its broad antimicrobial and anti‐inflammatory reputation [97]. In neurological contexts, folk use for memory enhancement has stimulated scientific interest in its anti‐Alzheimer potential. Phytochemically, *L. camara* contains a diverse spectrum of bioactive metabolites including triterpenoids, flavonoids, phenylpropanoids, iridoid glycosides, and essential oils rich in monoterpenes and sesquiterpenes, many of which demonstrate antioxidant, anti‐inflammatory, and cholinesterase inhibitory properties relevant to AD pathology [97]. In experimental evidence, a hydroethanolic leaf extract has been shown to significantly ameliorate scopolamine‐induced cognitive impairment in zebrafish and mice, improving spatial memory performance and reducing neuroinflammatory gene expression in hippocampal and cortical tissues; specifically, it downregulated IL‐1β, IL‐6, TNF‐α, and COX‐2 at doses of 10–100 mg/kg, suggesting strong antineuroinflammatory activity (Table 1). Additionally, essential oil fractions obtained via hydrodistillation demonstrated AChE inhibitory and antioxidant effects, supporting a dual mechanism of cholinergic enhancement and oxidative stress reduction (Table 1). Mechanistically, these actions align with AD pathology, where cholinergic depletion and neuroinflammation drive cognitive decline. Pharmaceutical interest in L. camara has therefore centered on its multitarget neuroprotective potential, including AChE inhibition, cytokine suppression, and free radical scavenging. However, despite these promising findings, clinical translation remains limited due to variability in phytochemical composition, dose‐dependent toxicity concerns (particularly hepatotoxic and cytotoxic diterpenes), and lack of standardized formulations. Recent studies highlight that while preclinical evidence supports its neuroprotective role, safety profiling, pharmacokinetics, and controlled human studies are still insufficient for therapeutic approval [100–102].

#### 3.1.6. *L. taraxacifolia* (Willd.) Amin ex C. Jeffrey

*L. taraxacifolia* (Asteraceae), widely referred to in West Africa as African wild lettuce or “efo yanrin,” is a perennial leafy herb that is traditionally consumed as a vegetable and is used medicinally across Nigeria, Ghana, Benin and neighboring regions. Ethnomedical practices describe its application for fever, wounds, digestive complaints, labor pain and metabolic disorders, particularly diabetes, reflecting long‐standing recognition of both its nutritional and therapeutic value [103]. Recent phytochemical analyses revealed a complex profile of polyphenols, flavonoids, tannins, saponins, alkaloids and sterols, alongside favorable proximate and mineral compositions that position the plant as a nutrient‐dense but underutilized leafy vegetable [104]. These chemical attributes have prompted increasing pharmacological interest, with studies demonstrating antioxidant, anti‐inflammatory, antidiabetic and hepatoprotective effects in vivo, thereby supporting its potential as a nutraceutical or phytopharmaceutical candidate [104, 105]. Emerging research has focused on neuroprotection, identifying properties relevant to AD. Preclinical findings have shown that aqueous and organic extracts of *L. taraxacifolia* mitigate biochemical and structural markers of neurotoxicity in rodent models, including aluminum‐ and cadmium‐induced brain injury and hypertension‐associated cognitive decline. Treated animals exhibit restored antioxidant enzyme activity, diminished glial activation, preserved neuronal morphology and improved performance in memory and behavioral assays, which is consistent with protection against the oxidative and inflammatory pathways implicated in AD [37, 106]. Enzyme‐based studies further suggest that the plant exerts mild inhibitory effects on cholinesterases, suggesting an additional mechanistic route by which *L. taraxacifolia* may support cholinergic function in neurodegenerative settings [37, 106]. Experimental validation has been supported by several recent in vivo investigations. Aqueous leaf extract (100–400 mg/kg) counteracts cisplatin‐induced oxidative neuronal injury in Wistar rats by increasing glutathione levels, reducing lipid peroxidation and preventing hippocampal and cortical degeneration, confirming its antioxidant‐driven neuroprotective potential [36]. In related models, doses of 100–200 mg/kg reduced markers of oxidative stress and attenuated astrocytic and microglial activation while simultaneously preserving neuronal structure and improving behavioral outcomes, demonstrating synergistic antioxidant and anti‐inflammatory actions relevant to AD pathogenesis [37]. Ethanol extracts administered at 274–822 mg/kg additionally ameliorated aluminum chloride‐induced deficits, enhancing motor coordination, cognitive flexibility and visuospatial function in AD [38].

#### 3.1.7. *L. inermis* L.

*L. inermis* (Lythraceae), commonly known as henna, is a medicinal shrub widely investigated for its neuroprotective potential against AD, a progressive neurodegenerative disorder characterized by Aβ aggregation, oxidative stress, and cholinergic dysfunction [73]. *L. inermis* is a glabrous, branched shrub or small tree (2–6 m tall) with opposite, entire, elliptic leaves, fragrant white or pinkish flowers arranged in large pyramidal cymes, and small brown capsules containing numerous seeds, features that are taxonomically characteristic of the Lythraceae family and documented in phytopharmacological literature [40]. The plant is widely distributed across tropical and subtropical regions including South Asia, the Middle East, and North Africa, thriving in arid and semiarid climates, which supports its extensive traditional use in countries such as India, Bangladesh, Iran, and Sudan*. L. inermis* has long been used in traditional medicine systems for treating skin disorders, inflammation, infections, and neurological conditions, with leaves and flowers particularly valued for their cooling, antimicrobial, and memory‐enhancing properties [40]. Phytochemical investigations have revealed a rich composition of bioactive constituents including naphthoquinones (lawsone), flavonoids, tannins, and triterpenoids, along with specific compounds such as β‐sitosterol, betulinic acid, oleanolic acid, and coumaroyl derivatives that contribute to its pharmacological profile, especially antioxidant and anticholinesterase activities relevant to AD pathology [107]. Notably, a novel compound, 1,2,4‐trihydroxynaphthalene‐2‐O‐β‐D‐glucopyranoside, isolated from the leaves, has demonstrated potent antioxidant and anti‐Aβ aggregation effects, directly targeting a key pathological hallmark of AD [107]. Experimental investigations on *L*. *inermis* demonstrate notable neuroprotective potential against AD through multiple in vivo and in vitro approaches. In a zebrafish Alzheimer’s model induced by sodium valproate, the aqueous extract prepared from the leaves and flowers exhibited dose‐dependent neurobehavioral improvements at concentrations of 15, 30, 250, and 500 μg, where treated organisms showed reduced anxiety‐like behavior, decreased neuronal degeneration, and improved motor coordination, alongside a significant enhancement in memory and learning performance. Complementing this, an ethanol extract of the leaves evaluated in an in vivo system at a dose of 25 mg/kg further substantiated the cognitive benefits of the plant, demonstrating marked improvements in learning and memory functions that were closely associated with its antioxidant activity, suggesting that mitigation of oxidative stress plays a central role in its mechanism of action. In another in vivo model using D‐galactose‐induced AD in Wistar rats, methanolic extracts of *L. inermis* seeds administered at 150 mg/kg significantly increased ACh levels while reducing AChE activity in the cerebral cortex, thereby indicating restoration of cholinergic neurotransmission, a key therapeutic target in Alzheimer’s pathology (Table 1). At the molecular level, phytochemical analysis has identified several active triterpenoids and sterols including β‐sitosterol, 3‐O‐β‐acetyloleanolic acid, 3‐O‐(Z)‐coumaroyl oleanolic acid, betulinic acid, and oleanolic acid, which contribute to the enzyme inhibitory properties of the plant. These compounds were further evaluated through in vitro AChE/BChE inhibition assays at concentrations of 1, 10, 20, and 40 μg/mL, where the extracts demonstrated potent and selective inhibition of BChE, with certain compounds exhibiting stronger inhibitory activity than others, highlighting their relevance particularly in later stages of AD where BChE activity becomes more prominent (Table 2). These pharmacological actions are largely attributed to its strong antioxidant capacity, which mitigates reactive oxygen species and prevents neuronal damage, as well as its ability to inhibit amyloid aggregation and improve synaptic function [107]. From a pharmaceutical perspective, *L. inermis* presents promising applications as a natural neuroprotective agent, particularly in the development of plant‐based anti‐Alzheimer drugs targeting multiple pathways such as oxidative stress, amyloidogenesis, and cholinesterase inhibition [40]. However, several limitations remain, including variability in phytochemical composition depending on geographical origin, lack of standardized dosing, insufficient clinical trials, and incomplete understanding of its molecular mechanisms, which hinder its translation into clinical therapeutics [40]. Furthermore, most current evidence is derived from preclinical models, necessitating well‐designed human studies to establish safety, efficacy, and pharmacokinetics before its integration into mainstream medicine [40]. It represents a valuable medicinal plant with multifaceted anti‐Alzheimer potential supported by emerging experimental evidence, yet further research is essential to fully harness its therapeutic applications in neurodegenerative disease management [39].

#### 3.1.8. *M. lutea* (Benth.) K. Schum

*M. lutea* (family Bignoniaceae), commonly known as the Nile tulip tree, is a medium‐sized ornamental and medicinal tree characterized botanically by opposite, pinnate leaves, trumpet‐shaped yellow flowers, and elongated pods, features typical of the genus and useful for species identification in pharmacognostic studies. *M. lutea* is widely distributed across tropical Africa and parts of Asia, thriving in savannahs, forest margins, and cultivated landscapes, which has facilitated its incorporation into traditional medicinal systems across diverse regions. Various parts of the plant including leaves, bark, and roots have been used in indigenous medicine for the treatment of inflammatory disorders, microbial infections, pain, and gastrointestinal conditions, suggesting a broad pharmacological relevance that aligns with its phytochemical diversity. Phytochemical investigations have revealed that *M. lutea* contains a rich array of bioactive compounds such as flavonoids, phenylpropanoid glycosides, terpenoids, lignans, quinones, tannins, and phytosterols, many of which are associated with antioxidant and neuroprotective activities relevant to neurodegenerative diseases. In the context of AD, which is characterized by cholinergic dysfunction, oxidative stress, and Aβ aggregation, these phytoconstituents are particularly significant because plant‐derived antioxidants and cholinesterase inhibitors have been widely recognized as promising therapeutic candidates [108]. Experimental evidence supports the anti‐Alzheimer potential of *M. lutea*, as ethanolic extracts of its leaves and flowers demonstrated strong antioxidant activity and significant inhibition of AChE, BChE, and Aβ aggregation in vitro (Table 1), indicating its multitarget neuroprotective effects. Further recent studies employing advanced analytical techniques such as UHPLC–ESI–TOF–MS have identified numerous secondary metabolites (over 60 compounds) and confirmed that flavonoid‐rich extracts exhibit superior antioxidant and anticholinesterase activities, in some cases comparable or superior to standard drugs like donepezil and rivastigmine [45]. Additional investigations have also demonstrated that *M. lutea* extracts can inhibit up to ∼77% of AChE activity and improve biochemical markers associated with cognitive function in experimental AD models, further validating its therapeutic relevance. Moreover, recent studies that the Nile tulip tree possesses in vitro anti‐Alzheimer, antioxidant, and cytotoxic properties, reinforcing its potential as a multifunctional medicinal plant for neurodegenerative disorders [109]. From a pharmaceutical perspective, *M. lutea* represents a promising source of lead compounds for the development of novel anti‐Alzheimer agents due to its multitarget mechanisms, including enzyme inhibition, free radical scavenging, and interference with amyloid pathology; such multitarget approaches are increasingly favored in AD drug discovery to address the complex etiology of the disease [108]. Several limitations hinder its clinical translation, including the lack of standardized extraction protocols, insufficient in vivo and clinical studies, variability in phytochemical composition due to environmental factors, and limited data on toxicity, pharmacokinetics, and long‐term safety. Additionally, while in vitro findings are promising, they do not always translate directly into clinical efficacy, emphasizing the need for rigorous preclinical and human studies.

#### 3.1.9. *M. vulgare* L.

*M. vulgare* (white horehound), a perennial Lamiaceae herb characterized by woolly stems, gray‒green wrinkled foliage and pale flowers, has long been used in traditional European and Mediterranean medicine. Historical and modern ethnobotanical records describe its use as an expectorant, digestive bitter and wound‐healing agent, as well as a remedy for respiratory disorders, hypertension and diabetes, highlighting its broad relevance in community pharmacopoeias [110, 111]. Contemporary phytochemical investigations reveal a profile dominated by labdane‐type diterpenes, especially marrubiin together with phenolic acids, flavonoids and an expanding group of phenylethanoid glycosides; collectively, these constituents provide a biochemical rationale for the plant’s reproducible antioxidant and anti‐inflammatory actions in vitro and in vivo [43, 110]. This traditional interest has converged with neuropharmacology, prompting the evaluation of *Marrubium* extracts for AD. Standardized preparations and isolated fractions demonstrate inhibitory activity against AChE, robust free‐radical scavenging capacity and attenuation of oxidative damage markers, whereas rodent experiments using scopolamine or related dementia models consistently report improved cognitive performance, restoration of cholinergic function and reduction of lipid peroxidation in brain tissue [42, 43]. Current mechanistic evidence suggests that *Marrubium* and associated polar phenolics act through complementary pathways, including mild cholinesterase inhibition, enhancement of endogenous antioxidant enzymes (GSH, catalase and SOD), modulation of neuroinflammatory mediators and protection of synaptic regulatory protein mechanisms that collectively may limit conditions favoring Aβ‐associated neurotoxicity [43, 112]. From a translational standpoint, *M. vulgare* represents a promising botanical reservoir for AD research. Standardized extracts enriched with phenylethanoid glycosides could be used as nutraceuticals to target early oxidative and cholinergic deficits, whereas *Marrubium* provides a structurally tractable scaffold for small‐molecule development. However, achieving clinical relevance will require rigorous dose optimization, comprehensive ADME and safety profiling, and improved control of phytochemical variability across plant populations, which are critical steps for moving *Marrubium*‐derived products into well‐designed preclinical and eventual clinical pipelines [110]. Recent experimental studies further reinforce this neuroprotective profile. In scopolamine‐challenged Wistar rats, a 200 mg/kg water extract improved recognition memory, restored hippocampal ACh and noradrenaline levels, normalized p‐CREB signaling and increased BDNF and Bcl‐2 expression while simultaneously reducing oxidative stress, indicating synergistic cholinergic, neurotrophic and antioxidant effects [42]. Hydroethanolic extracts displayed significant in vitro inhibition of AChE, BChE and tyrosinase across the 100–1000 μg/mL range, supporting their potential as multitarget botanical candidates for AD intervention [43]. Aqueous and methanolic whole‐plant extracts also enhanced memory performance in mice at 700–1400 mg/kg by modulating glutamatergic neurotransmission and counteracting oxidative injury in a scopolamine‐induced dementia model [44].

#### 3.1.10. *P. minor* Opiz

*P. minor*, widely known as kesum in Southeast Asia, is an aromatic Polygonaceae herb whose narrow, fragrant leaves are commonly used both as a culinary flavoring and as a traditional remedy for digestive discomfort, headaches, scalp conditions and general fatigue. Ethnobotanical documentation across Malaysia, Indonesia and neighboring regions consistently reinforces its dual role as food and medicine [113, 114]. Its phytochemical profile is dominated by phenolics and flavonoids, particularly quercetin‐derived polyphenols, together with volatile terpenoids, which are responsible for its characteristic aroma. These metabolites underpin the strong antioxidant, anti‐inflammatory and free‐radical scavenging capacities repeatedly demonstrated in recent analytical and pharmacological evaluations [115, 116]. Because these activities converge on biological processes central to AD oxidative stress, neuroinflammation and cholinergic dysfunction, *P. minor* has gained attention as a potential multitarget botanical for neurodegenerative disorders [46, 115]. Emerging preclinical studies provide supportive evidence. In a chronic cerebral hypoperfusion model that mimics aspects of vascular cognitive impairment and contributes to AD pathology, standardized leaf extracts improved recognition and spatial memory, increased hippocampal ACh and GABA levels, and produced metabolic signatures involving retinol, histidine and glucuronate pathway changes consistent with enhanced neuronal resilience and cholinergic support [46]. Complementary in vitro work using SH‐SY5Y neuroblastoma cells revealed that ethanolic extracts protect against hydrogen peroxide–induced oxidative injury through activation of the Nrf2/ARE axis, modulation of NF‐κB/IκB and MAPK signaling, and stabilization of ACh levels following oxidative stress [47]. Isolated compounds such as Polygonumins B and C further demonstrate potent AChE and BChE inhibitory activity at 100 μg/mL alongside high antioxidant capacity, reinforcing the potential relevance of the plant for symptomatic and disease‐modifying AD targets [76]. Complementary behavioral and stress models report antidepressant‐like and antistress effects of aqueous and ethanolic extracts, supporting a broader neuropsychotropic profile that could be therapeutically relevant in mixed dementia syndromes where mood and cognition interact [117]. Importantly, translational steps have progressed beyond the bench: a randomized, double‐blind, placebo‐controlled trial of a commercial *P. minor* extract (Biokesum) in older adults with mild cognitive impairment (MCI) examined cognition, mood and biomarkers over six months and provided early clinical data on safety, biomarker signals and cognitive endpoints, an encouraging bridge from preclinical promise to human studies, although larger, disease‐specific AD trials remain necessary to establish efficacy, dosing and mechanism [118].

#### 3.1.11. *P. dulce* (Roxb.) Benth.

*P. dulce* (commonly known as Manila tamarind, Madras thorn, or camachile) is a thorny leguminous tree widely naturalized across tropical regions and is characterized by coiled pods, bipinnate leaves, and small powder‐puff flowers. Ethnomedical records consistently highlight the broad therapeutic use of its leaves, bark, seeds, and fruits, which have been applied orally and topically in traditional systems for gastrointestinal disturbances, respiratory complaints, wound healing, pain, fever, and metabolic disorders, particularly diabetes [119, 120]. Over the past decade, phytochemical investigations have revealed diverse metabolite profiles, including flavonoids, tannins, saponins, alkaloids, sterols, triterpenoids, and abundant phenolics, supporting its antioxidant activity and providing a biochemical basis for its long‐standing medicinal relevance and potential for standardized extract development [73, 105]. The growing interest in AD has positioned *P. dulce* as a promising botanical candidate because its pharmacological actions intersect with key AD mechanisms. Preclinical studies have reported significant antioxidant effects, attenuation of cholinergic dysfunction, and modulation of monoamine systems, with several extracts inhibiting AChE and influencing dopamine noradrenaline pathway mechanisms intimately linked to cognitive preservation in AD [50, 119]. These findings have stimulated discussion of the translational potential of *P. dulce*, both as a standardized botanical product enriched in phenolics and flavonoids for nutraceutical or adjunctive therapeutic use and as a reservoir of lead compounds (particularly flavonoid and triterpenoid scaffolds) with the potential for medicinal‐chemistry optimization to increase BBB permeability and target specificity [120, 121]. However, direct experimental evidence demonstrating BBB penetration of *P. dulce* or its specific phytoconstituents remains unavailable, and current assumptions are largely extrapolated from the known pharmacokinetic behavior of structurally related compounds. Notably, *P. dulce* is rich in flavonoids and phenolic compounds, for which increasing experimental evidence indicates measurable brain bioavailability. For instance, phenolic compounds have been shown to cross the BBB and accumulate in brain tissue via transporter‐mediated and passive diffusion mechanisms, although often after metabolic transformation [122]. Similarly, pharmacokinetic studies of flavonoids demonstrate that BBB permeability is influenced by lipophilicity, molecular size, and interaction with efflux transporters, with several compounds detected in brain endothelial models and in vivo systems [123]. Despite this, quantitative brain distribution data (e.g., brain/plasma ratios, Kp, brain values) specific to *P. dulce* extracts or isolated compounds are currently lacking, highlighting a critical translational gap. Furthermore, the BBB restricts ∼98% of small‐molecule drugs, underscoring the challenge of achieving therapeutically relevant CNS exposure without optimized delivery systems [124]. To overcome this limitation, validated strategies such as nanocarrier‐based delivery systems (e.g., liposomes, solid lipid nanoparticles) or permeability enhancers have been proposed to improve CNS bioavailability of plant‐derived compounds [125]. Supporting evidence from recent experimental models strengthens this rationale: ethanolic leaf extracts improved scopolamine‐induced memory impairment in rats at doses of 200–400 mg/kg, enhancing locomotor and cognitive performance, increasing catalase activity, reducing malondialdehyde (MDA), and decreasing brain AChE activity [48]. Methanolic extracts of aerial parts demonstrated similar neuroprotective effects on aluminum chloride (AlCl_3_)‐induced cognitive dysfunction in zebrafish and mice, improving escape latency and target quadrant retention while normalizing AChE activity and restoring antioxidant enzymes (SOD, CAT, and GSH) with concurrent reductions in MDA [49]. In vitro assays further revealed that potent AChE inhibition (IC_50_ 19.23 μg/mL) was accompanied by antioxidant activity and modulation of the cholinergic and monoaminergic systems, suggesting multifaceted neuroprotective potential [50]. Additional in vivo work in scopolamine‐induced AD models using methanolic leaf extracts at 100–500 mg/kg further revealed dose‐dependent improvements in behavioral and biochemical markers. Collectively, these convergent data highlight *P. dulce* as an emerging botanical resource with mechanistic relevance to AD and a strong foundation for future preclinical standardization and therapeutic development [50]. Therefore, while the neuroprotective effects of *P. dulce* in preclinical models are promising, future studies must incorporate pharmacokinetic profiling, brain distribution studies, and advanced delivery approaches to substantiate its CNS therapeutic potential.

#### 3.1.12. *S. aristata* Aucher ex Benth.

*S. aristata* (family Lamiaceae) is a perennial sage species that has long been incorporated into traditional medical practices across the Middle East and Mediterranean, where preparations from its aerial parts have been used to manage respiratory ailments, digestive discomfort, and nervous system disorders. These ethnomedicinal patterns align with the broader therapeutic profile of the *Salvia* genus, which is widely recognized for its carminative, antiseptic, sedative, and mood‐modulating actions, providing context for its traditional application in age‐related cognitive complaints [126]. Recent phytochemical studies have shown that *S. aristata* contains diverse mixtures of phenolics, flavonoids, and terpenoids. Detailed GC–MS analyses revealed a high abundance of sesquiterpene hydrocarbons, particularly β‐trans‐caryophyllene, along with notable phenolic compounds and the flavone glycoside linariin, a profile that supports its antioxidant, metal‐chelating, and enzyme‐modulating properties relevant to neuroprotection [52]. Building on this chemical foundation, emerging preclinical work has demonstrated that hydroalcoholic extracts of *S. aristata*, and to a lesser degree its dichloromethane fractions, inhibit AChE, neutralize free radicals, and chelate metal ions in vitro. Isolated linariin reproduces several of these effects, indicating that the plant may act through both multitarget phytochemical combinations and single active molecules, converging on key mechanisms implicated in AD, including oxidative stress, cholinergic dysfunction, and metal imbalance [51]. These mechanistic findings translate into coherent pharmaceutical potential, and standardized extracts or phenolic‐rich fractions could be developed as oral nutraceuticals or adjunct phytopharmaceuticals offering symptomatic support through reversible AChE inhibition and complementary antioxidant or metal‐chelating actions. The essential oil composition adds further translational value, suggesting its use in volatile‐based formulations or nanoemulsions designed to enhance the delivery of bioactive terpenoids and flavonoids across the BBB [52]. However, direct experimental evidence supporting BBB permeability of *S. aristata* or its isolated constituents remains limited. A recent in vitro and in vivo investigation of *S. aristata* extracts confirmed neuroprotective and cognitive‐enhancing effects but explicitly highlighted the absence of pharmacokinetic and brain bioavailability data, emphasizing the need for dedicated BBB permeability and distribution studies [127]. In the absence of species‐specific data, indirect insights can be drawn from mechanistically related *Salvia* species. For example, an in vitro BBB model (MDCK‐MDR1) demonstrated that key diterpenoids from *Salvia miltiorrhiza* exhibit low apparent permeability (P_app < 1 × 10^−6^ cm/s) and are subject to P‐glycoprotein–mediated efflux, significantly limiting brain penetration unless transporter inhibition strategies are employed [51] although *S. aristata* contains neuroactive phenolics and flavonoids, its BBB penetration should not be assumed. Instead, it likely depends on compound‐specific physicochemical properties and transporter interactions, and may require formulation‐based strategies to achieve therapeutically relevant brain exposure. Experimental studies reinforce this potential: Hydroalcoholic and dichloromethane extracts of the aerial parts improved cell viability and demonstrated strong antioxidant and metal‐chelating effects in PC12 cells at 2.5–20 μg/mL, with corresponding improvements in memory performance in scopolamine‐treated rats at 400 mg/kg [51]. Similarly, essential oils obtained by hydrodistillation exhibited selective BChE inhibition and protected PC12 cells from oxidative insult at concentrations of 63.5–500 μg/mL, further underscoring its neuroprotective potential [52]. Moreover, the broader evidence from related *Salvia* species—several of which have shown cognitive benefits and neuroprotective outcomes in animal models and early human studies supports the rationale for advancing *S. aristata* toward formal preclinical development, using *S. officinalis* and allied species as methodological benchmarks for extract standardization, toxicological evaluation, and dose optimization [128, 129].

#### 3.1.13. *T. foenum-graecum* L.

*T. foenum-graecum* (fenugreek), a widely cultivated member of the Fabaceae family, has long been incorporated into traditional medical systems across South Asia, the Middle East and parts of Europe, where its seeds and leaves are used as carminative agents, digestive tonics, galactagogues and remedies for general weakness. Ethnobotanical documentation continues to highlight fenugreek for conditions related to metabolism, aging and cognitive decline, reflecting enduring beliefs that the plant enhances memory and vitality [130]. Over the past decade, phytochemical studies have mapped a diverse portfolio of bioactive constituents, including galactomannans; steroidal saponins, such as diosgenin, trigonelline, and 4‐hydroxyisoleucine; and a wide array of flavonoids and phenolic compounds that collectively underpin its systemic actions and form mechanistic links to neuroprotection through antioxidant, anti‐inflammatory, antiamyloid and insulin‐sensitizing pathways [131, 132]. These findings have also informed translational work aimed at different development routes: Soluble fiber and galactomannan fractions are explored as nutraceuticals that target metabolic dysfunction (a known risk contributor to AD), whereas small molecules such as trigonelline and diosgenin are being evaluated as BBB‐permeable leads with the potential to modulate neuronal resilience and synaptic integrity [133, 134]. Preclinical research evidence that fenugreek extracts and purified constituents influence multiple Alzheimer‐relevant mechanisms. In vitro and in vivo models have shown that fenugreek attenuates Aβ‐induced cytotoxicity, limits oxidative damage, reduces proinflammatory mediator levels, inhibits AChE activity and improves behavioral performance in learning and memory tasks via increased antioxidant defenses, reduced neuroinflammation and the repair of insulin signaling disruptions in the brain [135, 136]. Rodent work further reinforces these outcomes: Whole‐seed preparations (1.0 g/kg) ameliorated diabetes‐associated cognitive impairment in T‐maze and Morris water maze tasks and prevented neuronal loss in associated histological analyses [53]. Methanolic seed extracts (200 mg/kg) improved acquisition and retention in scopolamine‐based amnesia models, with behavioral effects approaching those of piracetam and supported by biochemical evidence of AChE inhibition [54]. Among the isolated compounds, trigonelline (10 mg/kg) demonstrated notable efficacy in 5XFAD mice by enhancing recognition and spatial memory, reducing axonal degeneration and showing clear brain penetration, with creatine kinase‐B identified as a putative molecular target mediating axonal repair [74]. Diosgenin, evaluated at 1–10 mg/kg in the same transgenic model, similarly promoted axonal regeneration, reversed structural deterioration and restored memory performance, positioning it as a compelling neurodegenerative candidate for AD [75].

#### 3.1.14. *W. somnifera* (L.) Dunal

*W. somnifera* (ashwagandha; family Solanaceae) is a well‐known medicinal shrub whose roots and leaves have been used for centuries in Ayurvedic and South Asian medical systems to treat fatigue, insomnia, memory decline and conditions resembling age‐related cognitive impairment. Ethnobotanical surveys and contemporary reviews consistently describe the plant as a rasayana, a rejuvenating tonic traditionally valued for strengthening the nervous system and improving mental clarity across India and neighboring regions [137, 138]. Modern phytochemical research confirms that these traditional claims are supported by a rich chemical repertoire dominated by steroidal lactones known as withanolides, including withaferin A and withanolide A, along with alkaloids and sitoindosides. These compounds have been shown to regulate oxidative stress, inflammatory cascades, cholinergic transmission and proteostasis, providing mechanistic links to pathways central to AD pathology [139]. Across preclinical AD models, both whole‐plant extracts and those isolated withanolides consistently demonstrated robust neuroprotective activity. In transgenic and toxin‐induced rodent models, ashwagandha reduces Aβ accumulation and tau pathology, enhances the expression of synaptic proteins and long‐term potentiation, increases the expression of neurotrophic factors such as BDNF, and suppresses microglial activation and the production of proinflammatory cytokines. These changes correspond with significant improvements in learning and memory across behavioral tasks [56, 140]. Mechanistically, these benefits arise from multimodal effects, including antioxidant activity, activation of the Nrf2 pathway, inhibition of NF‐κB signaling, modulation of AChE in certain preparations, and support of neuronal survival pathways, all of which align with current therapeutic targets under investigation for both symptomatic and disease‐modifying AD treatments [139, 141]. Therefore, increasing pharmaceutical interest has focused on developing standardized root extracts enriched with ethanol fractions as oral nutraceuticals or adjunct phytopharmaceuticals. Recent formulation studies have evaluated strategies to increase bioavailability and brain penetration, including comparisons of leaf versus root extracts, nanoparticle carriers, and combination approaches designed to strengthen target engagement within the central nervous system [141, 142]. Although clinical evidence in confirmed AD remains preliminary, several trials in older adults and individuals with MCI reported improvements in memory, attention and stress‐related biomarkers, suggesting translational potential while underscoring the need for disease specific, adequately powered trials before any efficacy claims can be established [142, 143]. Safety assessments indicate that standardized ashwagandha preparations are generally well tolerated at the doses used in human studies; however, variability in extract composition, dosing strategies and study methodology remain significant barriers to regulatory approval and clinical standardization. As a result, a clear path forward involves rigorous preclinical and clinical translation using well‐characterized extracts, validated biomarkers and controlled trials to determine whether the promising mechanistic and behavioral effects observed in animal models are reproducible in human AD populations [137, 144]. Recent experimental data further support these prospects. Aqueous root extract improved locomotor behavior, recognition memory and spontaneous alternation in a thioacetamide‐induced hepatic encephalopathy rat model at 200–400 mg/kg while reducing brain MDA and iNOS levels, increasing GSH, and inducing Nrf2 and HO‐1 with concomitant downregulation of NF‐κB/MAPK signaling [48]. Methanolic root extract at similar doses enhances cognitive performance, reduces the amyloid plaque burden in the cortex and hippocampus, restores oxidative balance (MDA, SOD, and GSH) and upregulates the Ca^2+^ exchanger NCX3, attenuating neuroinflammation [56, 57]. At the molecular level, withaferin A (0.5–2 μM) lowered secreted Aβ_40_ levels and protected neuroblastoma cells from Aβ‐induced toxicity, improving neuronal morphology and providing direct evidence of antiamyloid activity [81]. In addition, withanolide A, withanolide B, withanoside IV and withanoside V exerted potent antiaggregation and cytoprotective effects in vitro at 10–100 μM by inhibiting Aβ_1-42_ aggregation, decreasing oxidative stress and enhancing cell survival in Aβ‐challenged neuronal cultures [82].

### 3.2. Bioactive Phytoconstituents of Medicinal Plants

Numerous in vivo and in vitro studies have highlighted the potential of various commonly consumed medicinal plant‐based foods for combating AD. These plants are rich in bioactive compounds that exhibit significant pharmacological activities, including AChE inhibition, antioxidant and anti‐inflammatory effects, neuroprotection, modulation of Aβ toxicity, and regulation of neuronal pathways. While many of these compounds are described as multitarget agents, in several cases the reported mechanisms originate from separate experimental contexts rather than being validated simultaneously. These phytoconstituents found in various medicinal plant‐based foods that show benefits against AD are detailed in Table 2, with their chemical structures represented in Figure 3.

FIGURE 3Chemical structure of bioactive phytoconstituent of medicinal plant.

**Figure:**
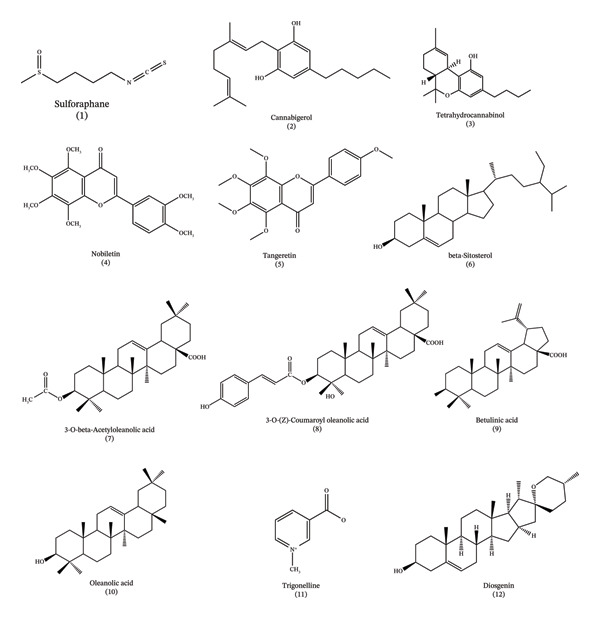

**Figure:**
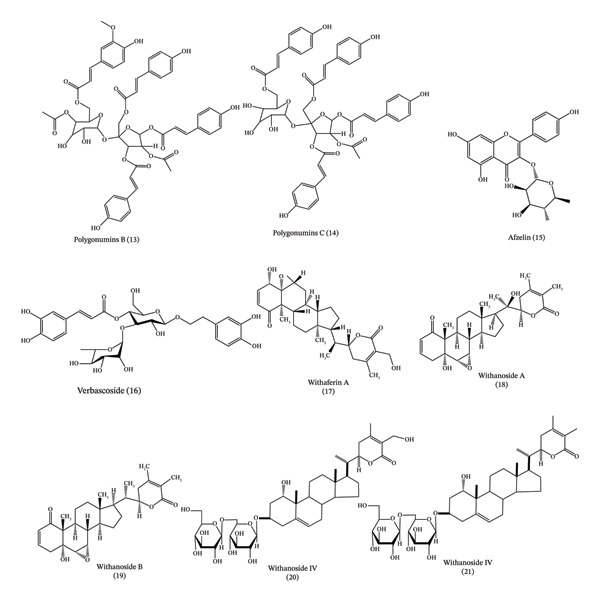

#### 3.2.1. Integrated Mechanistic Framework of Phytochemical Action in AD

Although oxidative stress, neuroinflammation, Aβ aggregation, tau hyperphosphorylation, and cholinergic dysfunction are often described as distinct pathological features of AD, these processes are now recognized as highly interconnected and mutually reinforcing components of a complex pathological network [6, 7]. Oxidative stress is considered a central upstream driver that activates redox‐sensitive transcription factors such as nuclear factor kappa B (NF‐κB), thereby promoting neuroinflammatory signaling and cytokine release. This inflammatory cascade further enhances amyloidogenic processing and tau phosphorylation through activation of kinases such as glycogen synthase kinase‐3β (GSK‐3β) and mitogen‐activated protein kinases (MAPKs), accelerating neuronal dysfunction [10]. In parallel, Aβ accumulation contributes to mitochondrial dysfunction and excessive ROS generation, which amplifies oxidative damage and sustains neuroinflammatory responses. Tau pathology further disrupts cytoskeletal stability and axonal transport, exacerbating synaptic failure and neuronal degeneration. Cholinergic dysfunction, characterized by reduced ACh levels and increased AChE activity, not only contributes to cognitive decline but also interacts with oxidative and inflammatory pathways, further aggravating disease progression [6, 7]. Importantly, evidence synthesized in this review indicates that plant‐derived phytochemicals act on multiple interconnected pathways rather than isolated targets (Table 2). For example, sulforaphane activates the Nrf2/ARE antioxidant pathway while suppressing NF‐κB‐mediated inflammation [66]; nobiletin and tangeretin modulate Aβ production, oxidative stress, and cholinergic signaling [71]; trigonelline and diosgenin improve neuronal metabolism and axonal integrity [74]; and withanolides reduce Aβ aggregation, oxidative stress, and neuroinflammation [81].

Figure 3 shows schematic diagram illustrating the anti‐Alzheimer’s mechanisms of action of various phytoconstituents found in medicinal plant.

### 3.3. Clinical Trials of Medicinal Plants for the Treatment of AD

Although a growing number of medicinal plants have advanced from preclinical investigations to human studies, the overall clinical evidence remains limited in scale, heterogeneous in design, and often exploratory in nature (Table 3). Most trials employ randomized, double‐blind, placebo‐controlled designs, which represent a methodological strength; however, several critical limitations including small sample sizes, short intervention durations, variability in outcome measures, and inconsistent standardization of botanical preparations restrict the robustness and generalizability of findings. Among the most extensively investigated botanicals, *Bacopa monnieri* has demonstrated relatively consistent cognitive benefits. A triple‐blinded, randomized, placebo‐controlled trial involving 62 patients with MCI reported improved cognitive performance after two months of supplementation at 160 mg [145]. Despite its rigorous design, the relatively small sample size and short intervention period restrict the generalizability of these findings and preclude conclusions regarding long‐term disease modification. Furthermore, the study primarily relied on neuropsychological test scores rather than validated AD‐specific clinical endpoints. Similarly, a randomized controlled trial evaluating a combined formulation of *B. monnieri*, *Panax quinquefolius*, and whole coffee fruit extract in 52 healthy adults demonstrated improvements in delayed recall and positive affect over 42 days [146]. However, the inclusion of cognitively healthy participants limits its translational relevance to AD populations. Additionally, the multicomponent nature of the formulation makes it difficult to attribute observed effects to individual constituents, thereby complicating mechanistic interpretation. Other plant‐derived interventions have been explored in specific clinical contexts. A double‐blind, randomized, placebo‐controlled pilot trial of *Boswellia serrata* in 80 patients with traumatic brain injury (TBI) showed significant cognitive improvements after three months [147]. While these findings suggest anti‐inflammatory mechanisms may support cognitive recovery, extrapolation to AD is limited due to differences in underlying pathology between TBI and AD. The study’s pilot nature and relatively short duration further underscore the need for larger, disease‐specific trials. Cannabinoid‐based therapies have also been investigated for AD‐associated symptoms. A phase II randomized, placebo‐controlled trial assessing a low‐dose combination of *C. sativa*‐derived THC and CBD over 26 weeks reported favorable safety and efficacy outcomes in patients with AD‐related dementia [148]. Nonetheless, the primary endpoints were largely focused on neuropsychiatric symptoms rather than core cognitive decline, and concerns remain regarding long‐term safety, dose optimization, and regulatory variability. In contrast, not all plant‐derived interventions have yielded positive or consistent clinical outcomes, underscoring the challenges of translating preclinical promise into therapeutic efficacy. A 36‐week randomized, double‐blind, placebo‐controlled trial of *C. reticulata* peel extract (400 mg daily) in 80 individuals with subjective cognitive decline reported no significant cognitive benefit compared to placebo [149]. This negative finding was largely attributed to a substantial placebo response, a well‐recognized limitation in early‐stage cognitive trials where subjective endpoints and participant expectations can obscure true treatment effects. Additionally, the reliance on subjective cognitive decline rather than clinically confirmed MCI or AD may have further reduced the sensitivity of outcome measures. In contrast, a randomized, double‐blind, placebo‐controlled study of *T. foenum-graecum* seed extract in 82 patients with mild‐to‐moderate memory impairment demonstrated significant improvements in memory performance, quality of life, and cardiovascular parameters after four months of treatment (500 mg/day) [150], While these findings are encouraging, the study’s moderate sample size and lack of standardized AD‐specific cognitive scales limit comparability with other trials. Moreover, the observed improvements in oxidative stress biomarkers (e.g., reduced MDA and increased total antioxidant capacity) suggest systemic rather than disease‐specific effects, raising questions about the extent to which these changes translate into long‐term neuroprotection. Similarly, *Polygonum minus* has shown potential multimodal benefits. In a six‐month randomized, double‐blind, placebo‐controlled trial involving 36 older adults with MCI, supplementation (250 mg twice daily) improved visual memory, mood, and biochemical markers such as BDNF and triglyceride levels [118]. However, the small sample size and limited statistical power reduce confidence in the robustness of these findings, and the absence of longitudinal follow‐up prevents assessment of sustained cognitive benefit or disease progression. Consistent with these findings, *W. somnifera* (ashwagandha) has demonstrated cognitive‐enhancing effects in a 60‐day randomized, double‐blind, placebo‐controlled trial involving 40 patients with MCI. Participants receiving 250 mg daily showed improvements in multiple cognitive domains, including immediate and working memory, attention, and processing speed. [151]. Despite these promising results, the short duration of the trial and relatively small cohort limit conclusions regarding long‐term efficacy and disease‐modifying potential. Across studies, a major limitation is the lack of standardization in botanical formulations, dosing regimens, and bioactive compound characterization, which complicates reproducibility and cross‐study comparisons. Furthermore, most trials focus on early cognitive impairment or non‐AD populations, with relatively few well‐powered studies conducted specifically in patients with clinically diagnosed AD. Long‐term safety data and assessments of disease progression remain largely absent. While early clinical evidence supports the cognitive‐enhancing and neuroprotective potential of several medicinal plants, the current body of research is insufficient to support definitive clinical recommendations. Future studies should prioritize larger, multicenter randomized controlled trials with standardized interventions, longer follow‐up periods, and validated AD‐specific endpoints to establish clinical efficacy and translational relevance.

**TABLE 3:** Clinical trials investigating the use of medicinal plants for preventing and treating AD.

Plant name	Study type	Administered material	Duration	Number of patients	Health condition of patients	Dose	Result	Ref
*Bacopa monnieri* (L.) Wettst. (Plantaginaceae)	A triple‐blinded, randomized, placebo‐controlled trial		2 months	62	Patients with mild cognitive impairment	160 mg	↑ cognitive performance	[145]
*Bacopa monnieri* (L.) Wettst, *Panax quinquefolius ginseng* (Araliaceae), and whole coffee fruit extract	A randomized, double‐blind, placebo‐controlled,	Tablet	42 days	52	Healthy adults	500 mg per day.	↑ positive affect and reliable gains in delayed recall after demanding working‐memory tasks.	[146]
*Boswellia serrata* Roxb. (Burseraceae)	Double‐blind, randomized, placebo‐controlled pilot clinical trial		3 months.	80	Patients with traumatic brain injury		↑ cognitive function	[147]
*Cannabis sativa* L. (Cannabaceae)	A Phase 2, randomized, double‐blind, placebo‐controlled, clinical trial	THC‐CBD (tetrahydrocannabinol‐ cannabidiol)	26 weeks		Patients with AD‐associated dementia	0.350 mg/THC and 0.245 mg/CBD	Effective and safe therapeutic option for AD‐related dementia.	[148]
*Citrus recticulata* (*Citrus* peel) (Rutaceae)	Randomized, double‐blind, parallel, monocentric, placebo‐controlled clinical trial	*Citrus* peel extract	36 weeks	80	Patients with cognitive decline	400 mg	Showed no cognitive advantage over placebo, likely due to a strong placebo response.	[149]
*Trigonella foenum-graecum* L. (Fabaceae)	Randomized, double‐blind, placebo‐controlled trial	Fenugreek seed extract	4 months	82	Patients with mild‐to‐moderate memory deficit.	500 mg	Shown promising effects on memory, quality of life, BP, serum MDA, and TAC levels	[150]
*Persicaria minor* Opiz (Polygonaceae)	Double‐blinded, randomized, and placebo‐controlled trial	Capsule	6 months	36	Patients with mild cognitive impairment (MCI)	250 mg twice daily	Improve visual memory, negative mood, BDNF, and triglycerides in older adults with MCI.	[118]
*Withania somnifera* (L.) Dunal (Solanaceae)	A randomized, double‐blind, placebo‐controlled study		60 days	40	Patients with mild cognitive impairment	250 mg daily	Improves immediate, general, and working memory, along with attention and processing speed in adults with MCI.	[151]

## 4. Herb–Drug Interactions in AD

Herb–drug interactions represent a critical but often underrecognized safety issue in elderly AD patients, who frequently receive multiple cholinesterase inhibitors, antidepressants, anticoagulants, and antipsychotics alongside self‐administered herbal medicines. Evidence indicates that concurrent use of herbal products with conventional drugs can alter pharmacokinetics (CYP450 modulation, P‐glycoprotein inhibition) and pharmacodynamics (additive sedative, cholinergic, or anticoagulant effects), thereby increasing risks of toxicity or therapeutic failure, particularly in polypharmacy settings common in geriatric AD care [152]. Systematic reviews further emphasize that older adults commonly use herbal medicinal products alongside prescription drugs, yet monitoring of clinically significant interactions remains insufficient in routine practice [153]. In relation to AD‐relevant medicinal plants, *W. somnifera* may enhance sedative effects when combined with benzodiazepines due to GABAergic potentiation, while also influencing CYP3A4‐mediated drug metabolism, raising concerns in multidrug regimens [154]. Similarly, *S. aristata* (sage‐related species) and other Lamiaceae members contain cholinesterase‐inhibiting phytochemicals that may potentiate donepezil or rivastigmine, increasing the risk of cholinergic adverse effects such as bradycardia and gastrointestinal distress [155]. *T. foenum-graecum* and *B. oleracea* may further complicate therapy through hypoglycemic or anticoagulant interactions, potentially interfering with comorbid diabetes or vascular medications commonly prescribed in AD populations. Additionally, herbal polypharmacy involving *C. sativa* and *M. vulgare* may exert CNS depressant effects, compounding sedation when combined with antipsychotics or anxiolytics. Overall, current evidence highlights a pressing need for systematic pharmacovigilance and clinician awareness regarding herb–drug interactions in AD, especially in elderly patients with multimorbidity and high medication burden, to prevent avoidable adverse outcomes and optimize therapeutic safety.

## 5. Toxicity, Long‐Term Safety, and Off‐Target Effects of Chronic Phytochemical Exposure in AD

Chronic exposure to phytochemicals used for AD management requires careful evaluation of toxicity, long‐term safety, and off‐target effects, particularly because many preclinical studies employ supra‐physiological doses that may not translate safely to humans. *W. somnifera* (L.) Dunal has demonstrated neuroprotective effects in AD models, but safety profiling indicates that clinically tested doses of 250–600 mg/day of standardized root extract are generally well tolerated, while higher experimental doses up to ∼1000 mg/kg in animals may produce mild central nervous system depression and thyroid hormone alterations, highlighting the importance of dose standardization in chronic use [156, 157] Importantly, a randomized controlled trial using 300 mg twice daily for 8 weeks showed cognitive benefits with no serious adverse events, supporting a relatively safe therapeutic window, although long‐term human safety beyond short clinical periods remains insufficiently characterized [157]. However, mechanistic reviews emphasize that bioactive withanolides may interact with CYP enzymes and neuroendocrine pathways, raising concerns about cumulative exposure and potential off‐target endocrine modulation during prolonged administration [158]. Similarly, *T. foenum-graecum* L. (fenugreek) has demonstrated anti‐amyloid and anti‐inflammatory activity in AD models, with neuroprotection observed at approximately 100 mg/kg/day in rodent cerebral hypoperfusion and toxin‐induced neurodegeneration studies, but translational evidence shows limited long‐term human safety data [159]. Fenugreek seed powder at dietary levels of 5–8 g/day has generally shown low toxicity in humans, though gastrointestinal discomfort and mild hypoglycemia are reported adverse effects, indicating possible metabolic off‐target risks in chronic administration, especially in elderly AD patients with comorbid diabetes. Furthermore, mechanistic reviews highlight that fenugreek’s active constituent trigonelline crosses the BBB and may modulate multiple neuronal signaling pathways, but its pharmacokinetic accumulation and long‐term neurotoxicity thresholds remain undefined, necessitating caution in chronic use [160]. For *C. sativa* L., emerging clinical evidence indicates potential cognitive and behavioral benefits in AD dementia at very low oral doses (e.g., THC 0.3–1.5 mg or THC/CBD balanced extracts), yet safety concerns remain due to central nervous system side effects including sedation, dizziness, and possible cognitive slowing at higher exposures [148, 161]. A randomized clinical trial of THC‐CBD extract reported a dose of approximately 0.35 mg THC daily over 26 weeks as well tolerated, with no major adverse events, but emphasized that long‐term neurocognitive safety beyond trial duration is still unknown [148]. Systematic reviews also note variability in cannabinoid formulations and dose‐dependent psychotropic effects, suggesting significant off‐target risks on attention, memory consolidation, and motor coordination with chronic exposure, particularly in elderly populations [162]. These medicinal plants demonstrate promising neuroprotective mechanisms against AD pathology, including amyloid modulation, antioxidant defense, and anti‐inflammatory effects; however, current evidence consistently highlights a critical gap in long‐term safety data, particularly regarding chronic exposure, cumulative neuroendocrine effects, and off‐target organ toxicity. Most clinical studies remain short‐term (4–12 weeks), and extrapolation to lifelong use in AD patients is not yet supported. Therefore, standardized dosing, pharmacokinetic profiling, and long‐term randomized controlled trials are essential before integrating these phytochemicals into sustained therapeutic regimens for AD.

## 6. Future Perspectives and Conclusion

Medicinal plants and their bioactive constituents represent a rapidly evolving frontier in AD therapeutics, offering a multidimensional approach capable of targeting oxidative stress, neuroinflammation, cholinergic dysfunction, amyloid–tau pathology, and synaptic decline. It is important to distinguish between experimentally validated multitarget mechanisms and those inferred from separate experimental models, as many reported pleiotropic effects have not been demonstrated concurrently within a single AD‐relevant system. In several cases, individual biological activities have been demonstrated in separate in vitro or in vivo studies rather than within a single integrated model, which may lead to an overestimation of true pleiotropic effects. For instance, antioxidant and anti‐inflammatory properties are often well supported by experimental evidence, whereas effects such as anti‐amyloid or tau‐modulating activities are sometimes extrapolated from non‐AD‐specific models or indirect assays. Therefore, the multitarget potential of phytochemicals should be interpreted with caution, and greater emphasis should be placed on studies that demonstrate concurrent modulation of multiple AD‐relevant pathways within unified experimental frameworks. Future research should prioritize integrative models to validate these combined effects and improve translational relevance. However, despite compelling preclinical evidence, the translational gap remains substantial. Most plant‐derived interventions have been evaluated *in* in vitro or small‐scale in vivo models, and only a limited number have advanced into well‐designed clinical trials. Future research must therefore prioritize large, multicenter randomized trials with standardized extracts, well‐defined dosing regimens, and biomarker‐based outcomes. To bridge the translational gap, a structured clinical roadmap is required. Prioritization of lead compounds should focus on phytochemicals with consistent in vivo efficacy and mechanistic validation, particularly sulforaphane, nobiletin, trigonelline, diosgenin, and withanolides, which demonstrate multitarget activity and translational potential. standardized outcome biomarkers should be incorporated to improve clinical relevance and trial sensitivity. Established and emerging biomarkers include CSF Aβ42/Aβ40 ratio, phosphorylated tau (p‐tau181 and p‐tau217), and neurofilament light chain (NfL), which have been extensively validated for diagnosis, disease progression, and therapeutic monitoring in AD [163, 164]. These biomarkers enable early detection and provide more sensitive endpoints than cognitive measures alone. Additionally, blood‐based biomarkers (e.g., p‐tau217 and Aβ42/40) are emerging as minimally invasive tools for large‐scale clinical trials and patient stratification [165]. Clinical translation will require standardization of phytochemical formulations, reproducibility across studies, and integration of biomarker‐driven trial designs, ensuring alignment with current AD drug development frameworks.

The heterogeneity of phytochemical composition due to geographic, seasonal, and extraction variabilities further necessitates the development of unified quality‐control protocols and pharmaco‐standardization strategies to ensure clinical reproducibility. This standardization should align with internationally recognized pharmacopeial frameworks such as the World Health Organization, which recommend the use of defined marker compounds, chromatographic fingerprinting, and validated analytical techniques (e.g., HPLC, GC–MS, DNA barcoding) to ensure identity, purity, and batch‐to‐batch consistency. Phytochemical variability is a well‐recognized challenge, as the composition of herbal products is significantly influenced by environmental factors such as climate, soil, and harvesting conditions, leading to inconsistencies in therapeutic efficacy and safety [166]. Moreover, marker‐based standardization and metabolomic fingerprinting approaches are increasingly recommended to ensure reproducibility, as the lack of well‐defined phytochemical markers remains a major obstacle to global acceptance and clinical translation of herbal medicines [167]. Despite the availability of regulatory guidelines, inconsistencies in raw materials, analytical methods, and reporting standards continue to hinder reproducibility and large‐scale clinical development, emphasizing the urgent need for harmonized global standards and rigorous quality‐control frameworks [167].

Another critical direction involves mechanistic deep profiling of active compounds via multiomics integration, including metabolomics, transcriptomics, and proteomics, to map their impact across interconnected AD pathways. Emerging evidence suggests that many phytoconstituents have pleiotropic benefits, such as sulforaphane, which activates Nrf2; trigonelline, which reverses axonal degeneration; and withanolides, which inhibits Aβ aggregation, indicating that poly‐target agents may be more advantageous than single‐pathway drugs.

Additionally, nanotechnology‐assisted delivery systems such as nanoemulsions, liposomes, and phytosomal formulations hold strong promise for enhancing BBB penetration, bioavailability, and pharmacokinetic stability of plant‐derived molecules. Concrete evidence supports this potential; for example, sulforaphane‐loaded polymeric nanoparticles demonstrated enhanced cellular uptake and significantly improved therapeutic efficacy compared to free sulforaphane, owing to improved intracellular delivery and stability [168]. Similarly, nobiletin‐loaded nanoemulsions exhibited improved physicochemical stability and significantly enhanced anti‐inflammatory activity relative to conventional formulations [169]. Nanocarrier systems further enhance such strategies; for instance, sulforaphane co‐delivered via liposomal nanocarriers significantly increased intracellular accumulation and synergistic activity when combined with conventional drugs compared to free‐drug formulations. These nanoformulations offer clear advantages, including controlled release, improved pharmacokinetics, and enhanced cellular uptake over conventional delivery systems [170] despite these advantages, safety and translational challenges remain, as nanocarriers may introduce variability in pharmacokinetics and uncertain biological interactions; for example, studies indicate that nanoemulsion‐based systems do not always improve brain delivery and their effectiveness depends strongly on drug‐specific properties [171]. Furthermore, issues such as formulation reproducibility, long‐term toxicity, and regulatory standardization continue to limit clinical translation.

Exploring synergistic combinations of phytochemicals with existing FDA‐approved drugs could also yield hybrid therapeutic strategies capable of slowing disease progression while minimizing toxicity. The interplay among neuroinflammation, insulin resistance, metabolic dysfunction, and AD suggests further opportunities to investigate medicinal plants that modulate neuroendocrine and gut–brain axis signaling. Long‐term dietary interventions incorporating plant‐based nutraceuticals may serve as preventive strategies in at‐risk populations. In parallel, genomic and pharmacogenomic studies should identify patient subgroups most likely to benefit from specific phytochemicals, paving the way for precision herbal therapeutics. Importantly, ethical considerations, the sustainable sourcing of medicinal plants, and the integration of traditional medical knowledge with modern pharmacology must guide the development of future therapies. Ethical considerations, the sustainable sourcing of medicinal plants, and the integration of traditional medical knowledge with modern pharmacology must guide the development of future therapies. Several medicinal plants discussed in this review are already subject to overharvesting pressure, including *W. somnifera*, *T. foenum-graecum*, *Salvia* spp., and *P. dulce*, primarily due to high commercial demand and reliance on wild or semiwild collection systems. For example, unsustainable harvesting of *W. somnifera* (ashwagandha) has been reported as a conservation concern in South Asia, necessitating regulated cultivation and good agricultural practices (GAP) to maintain supply and genetic diversity [172]. Similarly, *T. foenum-graecum* is increasingly promoted under controlled cultivation systems to reduce pressure on wild and marginal land populations while improving biomass yield through agronomic optimization [173]. To mitigate these issues, integrated strategies such as in situ conservation, ex situ germplasm preservation, and large‐scale cultivation are recommended, supported by biotechnological approaches like micropropagation and genetic resource banking. Overall, transitioning from wild harvesting to standardized cultivation enhances biodiversity conservation while improving phytochemical quality and supply stability for medicinal plant–based drug development [174].

In conclusion, current evidence indicates that medicinal plants and their bioactive constituents exert multitarget effects relevant to AD pathology, including antioxidant, anti‐inflammatory, cholinergic‐modulating, and anti‐amyloid actions. However, the strength of evidence varies considerably across experimental levels. Preclinical studies consistently support mechanistic and neuroprotective effects, while early‐phase clinical trials provide limited evidence of short‐term symptomatic improvement, particularly in cognitive and behavioral domains. In contrast, evidence for disease‐modifying efficacy in humans remains insufficient, and preventive effects should be considered preliminary, being largely derived from experimental and early clinical observations rather than long‐term clinical validation. Despite strong biological plausibility and encouraging translational signals, the clinical application of these phytochemicals is constrained by heterogeneity in formulations, limited large‐scale randomized trials, and insufficient long‐term safety data. Future progress will depend on standardized preparations, biomarker‐guided clinical endpoints, and rigorously designed multicenter trials to determine their true therapeutic and preventive value in AD.

**Nomenclature**
AD: Alzheimer’s disease
Aβ: Amyloid beta
Aβ_1-42_
: Amyloid beta peptide 1–42
ACh: Acetylcholine
AChE: Acetylcholinesterase
ARE: Antioxidant response element
APP/PS1: Amyloid precursor protein/presenilin‐1 (transgenic model)
BBB: Blood–brain barrier
BACE1: β‐site amyloid precursor protein cleaving enzyme 1
BCCAO: Bilateral common carotid artery occlusion
BChE: Butyrylcholinesterase
BDNF: Brain‐derived neurotrophic factor
BPSD: Behavioral and psychological symptoms of dementia
CBD: Cannabidiol
COX‐2: Cyclooxygenase‐2
CREB: cAMP response element‐binding protein
DPP‐4: Dipeptidyl peptidase‐4
ER: Endoplasmic reticulum
FDA: Food and Drug Administration
FoxO3a: Forkhead box O3
GABA: Gamma‐aminobutyric acid
GSH: Reduced glutathione
GSK‐3β: Glycogen synthase kinase‐3 beta
H_2_O_2_
: Hydrogen peroxide
HE: Hepatic encephalopathy
HO‐1: Heme oxygenase‐1
iNOS: Inducible nitric oxide synthase
IL‐1β: Interleukin‐1 beta
IL‐6: Interleukin‐6
LPS: Lipopolysaccharide
MCI: Mild cognitive impairment
MDA: Malondialdehyde
MAPK: Mitogen‐activated protein kinase
MANF: Mesencephalic astrocyte‐derived neurotrophic factor
MDA: Malondialdehyde
NCX3: Sodium/calcium exchanger 3
NF‐κB: Nuclear factor kappa B
Nrf2: Nuclear factor erythroid 2–related factor 2
PC12: Pheochromocytoma cell line
ROS: Reactive oxygen species
Ser: Serine
SIRT1: Sirtuin 1
TAA: Thioacetamide
TAC: Total antioxidant capacity
Tg: Transgenic
T2DM: Type 2 diabetes mellitus
THC: Tetrahydrocannabinol
TNF‐α: Tumor necrosis factor‐alpha
U251: Human glioma cell line

## Author Contributions

Conceptualization: Nawfal Hasan Siam; formal analysis: Nawfal Hasan Siam; funding acquisition: Nawfal Hasan Siam; investigation, resources, writing–original draft, review and editing: Nawfal Hasan Siam, Najibah Nasrin, Sharmily Saiyara, Hridoy Saha, Debashis Paul Deb, and Johirul Islam; supervision and reviewing: Nawfal Hasan Siam. All authors have participated sufficiently in the work.

## Funding

This research received no external funding.

## Disclosure

All authors read and approved the final manuscript and agreed to be accountable for all aspects of the work.

## Ethics Statement

The authors have nothing to report.

## Conflicts of Interest

The authors declare no conflicts of interest.

## Data Availability

The authors have nothing to report
